# CRISPR/Cas9 Screening Highlights PFKFB3 Gene as a Major Contributor to 5-Fluorouracil Resistance in Esophageal Cancer

**DOI:** 10.3390/cancers17101637

**Published:** 2025-05-12

**Authors:** Feng Xue, Hai Yang, Pengyan Xu, Shuman Zhang, Nathalie Britzen-Laurent, Li-Li Bao, Robert Grützmann, Christian Krautz, Christian Pilarsky

**Affiliations:** 1 Department of Surgery, Friedrich-Alexander-Universität Erlangen-Nürnberg (FAU) and Universitätsklinikum Erlangen, 91054 Erlangen, Germany; xuefeng.med@gmail.com (F.X.); xvpengyan@foxmail.com (P.X.); zhang_shu_man@163.com (S.Z.); nathalie.britzen-laurent@uk-erlangen.de (N.B.-L.); robert.gruetzmann@uk-erlangen.de (R.G.); christian.krautz@uk-erlangen.de (C.K.); 2 Department of Surgery, Juraklinik Scheßlitz, 96110 Scheßlitz, Germany; h.yang@gkg-bamberg.de; 3 Department of Medicine 1, Friedrich-Alexander-Universität Erlangen-Nürnberg (FAU) and Universitätsklinikum Erlangen, 91052 Erlangen, Germany; lili.bao@uk-erlangen.de

**Keywords:** esophageal squamous cell carcinoma, 5-Fluorouracil, CRISPR/Cas9, drug resistance, PFKFB3

## Abstract

Background: Esophageal cancer (EC) is the eighth most common cancer and the sixth most common cause of death worldwide. Esophageal squamous cell carcinoma (ESCC) comprises the majority of esophageal cancers globally, and 5-Fluorouraci (5-FU) is one of the commonly used chemotherapeutics for this type of cancer. Chemoresistance to drugs is a main obstacle in the successful treatment of this malignancy. Methods: In this study, we used the CRISPR/Cas9 screening method to determine the target gene related to 5-FU drug resistance in esophageal cancer. Results: Our research findings indicate that the loss of PFKFB3 can increase the resistance of different human esophageal squamous cell carcinoma cell lines to 5-FU through various pathways. Specifically, in KYSE-70 cells, loss of PFKFB3 can induce epithelial–mesenchymal transition (EMT) and prolong the S phase of the cell cycle, allowing cancer cells to evade the effects of 5-FU and develop resistance. In the KYSE-270 and KYSE-150 cell lines, loss of PFKFB3 can upregulate the expression of Slug and Mcl-1, indirectly regulate Chk1 and promote its autophosphorylation, which in turn inhibits apoptosis, thus counteracting the effects of 5-FU. Conclusions: Our research not only enriches our understanding of the biological characteristics of different ESCC cell lines but also provides new clinical insights for future personalized treatments. Assessing the status of PFKFB3 can help predict resistance to 5-FU in ESCC patients with different genetic backgrounds, allowing for more precise treatment planning. This personalized approach has the potential to improve treatment efficacy, reduce unnecessary drug use and side effects, and ultimately improve patient survival rates and quality of life.

## Simple Summary

Esophageal cancer is a major global health concern, particularly the subtype known as esophageal squamous cell carcinoma (ESCC), which is commonly treated with the chemotherapy drug 5-Fluorouracil (5-FU). However, resistance to 5-FU remains a significant challenge that limits treatment effectiveness. In this study, we used CRISPR/Cas9 gene-editing technology to identify genes involved in 5-FU resistance in ESCC cells. We found that the loss of the PFKFB3 gene enhances resistance to 5-FU through different molecular pathways in various cell lines. These findings offer new insights into the biological behavior of ESCC and may help predict which patients are more likely to develop resistance to 5-FU. This knowledge could support the development of more personalized and effective treatment strategies, ultimately improving patient outcomes.

## 1. Introduction

Esophageal cancer is the eighth most common cancer and the sixth most common cause of death worldwide [1], with more than 0.6 million new cases of esophageal cancer and 0.54 million deaths worldwide in 2020 [2]. The predominant histological types of esophageal cancer are squamous cell carcinoma (ESCC) and adenocarcinoma (EAC), and ESCC comprises the majority of esophageal cancers globally [2]. The incidence of these two histologic subtypes has significant geographical differences, with EAC being more prevalent in North America, Australia and European countries, and ESCC having a relatively high incidence in East Asia, South America and East Africa [2,3].

ESCC has a high degree of malignancy. Its treatment regimen is dependent on the tumor stage and the general status of patients. Patients who have locally advanced or disseminated disease at diagnosis should be treated with a systemic regimen [4] and 5-FU is one of the most commonly used chemotherapy drugs [5]. In recent years, immunotherapy has revolutionized the treatment landscape of esophageal cancer, particularly with the advent of immune checkpoint inhibitors targeting the PD-1/PD-L1 axis. The phase III KEYNOTE-590 clinical trial demonstrated that the addition of pembrolizumab to standard chemotherapy (5-FU and cisplatin) significantly improved overall survival and progression-free survival in patients with advanced esophageal cancer, establishing chemoimmunotherapy as the new first-line treatment standard [6,7]. Despite these advances, intrinsic and acquired resistance to chemotherapy remains a major clinical challenge, often compromising long-term therapeutic efficacy. Therefore, understanding the molecular mechanisms driving chemoresistance is essential for developing more effective and durable treatment strategies.

Over the past decade, as the most advanced gene editing technology, clustered regularly interspaced short palindromic repeats and CRISPR-associated protein9 (CRISPR/Cas9) have been widely used in the field of medical research [8,9,10]. This technology involves the use of artificially designed sgRNA (single-guide RNA) to identify target genomic sequences, guiding the Cas9 protein to effectively cut the DNA double strands and induce double-strand breaks (DSBs) [11,12]. After damaging the DNA, self-repair can take place, leading to mutations such as insertions or deletions in the target gene, ultimately modifying the target genomic DNA [11,12]. CRISPR/Cas9 screening is a large-scale genetic loss-of-function experimental approach designed to find a small number of target genes among large numbers of genetic sequences [10,12]. These screens use a single guide RNA (sgRNA) library of knockout genes across a population of cells by applying selective pressure and then identifying genes that influence many physiological effects [13]. Drug sensitivity and drug resistance are two of the major physiological responses that are frequently studied via CRISPR screening [13,14]. Next-generation sequencing (NGS) provides an efficient method for quantifying and comparing the frequencies of sgRNAs encoded in screened and control populations, allowing researchers to determine which gene knockouts yielded phenotypes associated with the screen [15,16].

The molecular basis of 5-FU resistance in esophageal cancer remains incompletely understood, largely due to the lack of systematic, high-throughput functional studies. Moreover, the intrinsic heterogeneity of esophageal cancer further complicates the identification of universal therapeutic targets. To address these challenges, we employed a CRISPR/Cas9 knockout screening approach to identify novel genes associated with 5-FU resistance. Among the candidate genes, PFKFB3, a critical glycolytic enzyme involved in metabolic reprogramming, emerged as a top hit. Subsequent validation experiments across three esophageal cancer cell lines confirmed its role in modulating 5-FU resistance through distinct mechanisms. These findings suggest that PFKFB3 may serve as a key mediator in drug-resistant esophageal cancer cells.

This study not only sheds light on the molecular landscape of 5-FU resistance but also highlights PFKFB3 as a potential therapeutic target for overcoming chemoresistance. Our findings offer valuable insights into the development of personalized treatment strategies and provide a foundation for future translational research in esophageal cancer therapy.

## 2. Results

### 2.1. Human Protein Kinase Library Screening Identifies PFKFB3 as Target Gene Involved in Esophageal Cancer Chemoresistance

**Figure 1:**
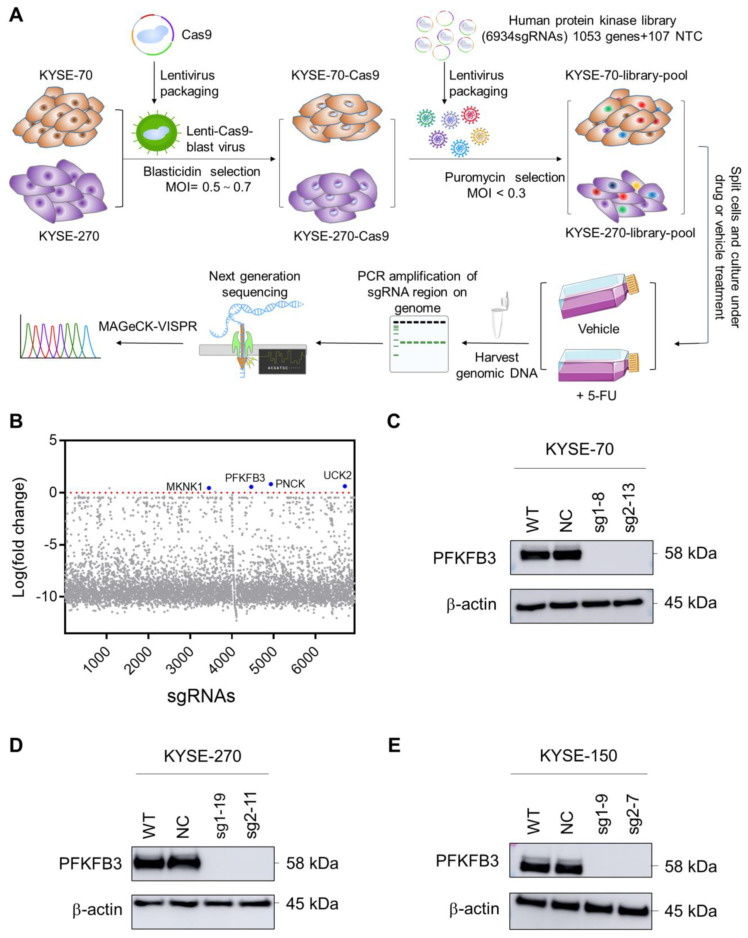
Human protein kinase library screen identified the PFKFB3 gene involved in esophageal cancer chemoresistance (**A**) Schematic outline of 5-FU drug resistance screening. (**B**) Identification of good sgRNA using MAGeCK-VISPR analysis. (**C**–**E**) PFKFB3 knockout KYSE-70, KYSE-270, and KYSE-150 cell lines were generated using CRISPR/Cas9 technology, and Western blot analysis was employed to confirm the efficiency of the PFKFB3 gene knockout.

To understand the role of protein kinase in the 5-FU resistance of esophageal cancer, this study conducted CRISPR/Cas9 protein kinase knockout library screening on two squamous esophageal cancer cell lines (KYSE-70 and KYSE-270). As shown in Figure 1A, we employed lentiviral transfection on these two cell lines and obtained stable cell lines expressing Cas9 through Blasticidin selection. Subsequently, the human protein kinase library was transfected into the Cas9 cell line. The library consists of 6934 sgRNAs, corresponding to 1053 genes and 107 NTCs (non-targeting controls). By controlling the MOI < 0.3, we aimed to transfect each cell with only one sgRNA. The library cells were divided into two groups after puromycin selection: The group with added 5-FU was designated the treatment group, and we named them KYSE-70-5FU and KYSE-270-5FU. The other group without added 5-FU served as the control group, named KYSE-70-3w and KYSE-270-3w. Each group was repeated three times. Then, a sufficient number of cells were collected, and genomic DNA was extracted for deep sequencing. Quality control and analysis of the deep sequencing results were performed using MAGeCK-VISPR [17,18], ultimately identifying genes associated with 5-FU resistance.

To ensure consistency among different datasets, we employed MAGeCK-VISPR for the unified analysis and processing of all raw deep sequencing results in fastq format. MAGeCK-VISPR, developed by Wei Li and others [17,18], is a powerful tool specifically designed for the analysis of CRISPR screening results. It encompasses comprehensive quality control (QC), analysis, and visualization workflows. The generated QC metrics include GC content, sequencing reads, mapped reads, zero sgRNAs, the Gini index, and more. In terms of exceeding the overall data quality, corresponding recommendations are provided, such as the distribution of the average base quality for each sample in each read, with peaks exceeding 30, and our results indicate values higher than 35 (Figure S1A). For the distribution of GC content, all samples from the same library should exhibit a similar pattern, as illustrated in our results (Figure S1B). The Gini index for each sample measures the degree of inequality between read counts. A value of 0.0 indicates that all sgRNAs have the same read count, while 1.0 represents maximum inequality. Our results show that the Gini index for the control group samples is around 0.1, indicating that the sgRNA counts are approximately equal across all control samples. However, post 5-FU treatment, the Gini index for the treatment group samples ranges from 0.7 to 1.0, indicating a significant inequality in sgRNA read counts, aligning with our anticipated hypothesis (Figure S1C). High-quality samples can also be indicated by a low count of missing sgRNAs. (Figure S1D). Due to the substantial cell death caused by 5-FU, the sgRNAs they carry are also depleted. Only a small population of cells resistant to 5-FU survives, and their corresponding sgRNAs are counted.

The results of the MAGeCK-VISPR analysis show 105 genes with *p* < 0.05 in the list of positive selection; however, the LFC (log fold change) value were all less than 0. (Table S1) We speculate that this may be due to the intensity of our screening conditions being too strong, which resulted in the loss of most of the sgRNAs. The list also shows that only four genes had corresponding good sgRNA. We further examined the list of sgRNAs in Table S1. Four of the genes that corresponded to LFC > 0 and FDR (False Discovery Rate) < 0.5 were PNCK, UCK2, PFKFB3, and MKNK1 (Figure 1B).

### 2.2. Knockout of PFKFB3 Enhances 5-FU Resistance in Human Esophageal Cancer Cell Lines

To explore the relationship between PFKFB3 and 5-FU resistance, we initially knocked out PFKFB3 in three human esophageal cancer cell lines (KYSE-70, KYSE-270, KYSE-150). To exclude any potential effects of the CRISPR technology on drug resistance, we included a negative control (NC) group alongside the wild-type (WT) group for each cell line. Furthermore, we employed two different sgRNAs to target the knockout of the PFKFB3, designated as sg1 and sg2.

Knockout efficiency was validated via Western blot (Figure 1C–E) and DNA sequencing (Table 1 and Figure S2A–F). The sequencing results provide information about the types of mutations. These mutation types can include point mutations, which are changes involving single nucleotides, insertions, or deletions. The results demonstrate that some monoclones exhibit a single form of mutation, whereas others display two distinct types. For instance, the KYSE-70-sg1-8 mutation type is characterized by the insertion of a 2 bp base (Table 1 and Figure S2A), while KYSE-270-sg2-11 has two mutation types, the insertion and deletion of 1 bp base (Table 1 and Figure S2F).

**Figure 2:**
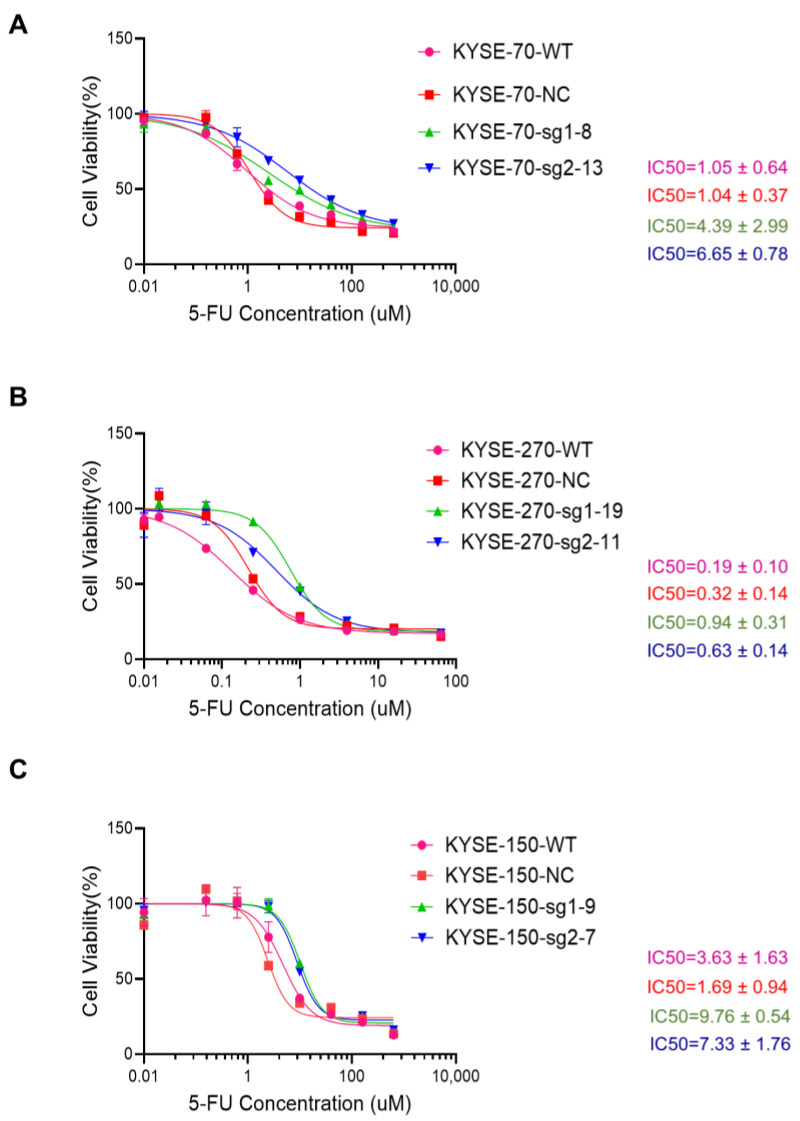
Knockout of PFKFB3 enhances 5-FU resistance in human esophageal cancer cell lines. (**A**–**C**) Dose–response curves of esophageal cancer cell lines treated with 5-FU and their corresponding IC50 values. Cell viability was assessed 72 h post-treatment. IC50 values were calculated using nonlinear regression in GraphPad Prism. Data are presented as the mean ± standard deviation (SD) from three independent experiments.

Following the validation of the PFKFB3 knockout cell lines, the cells were subjected to a further 3 days of exposure to 5-FU. The corresponding IC50 values were calculated and compared to observe the changes in their response to 5-FU before and after PFKFB3 knockout. The results show that the IC50 value of KYSE-70 WT and NC cells is approximately 1 µM, while that of PFKFB3 knockout is between 4 and 7 µM, representing a nearly 4–6-fold increase (Figure 2A). The IC50 of the WT and NC cells of KYSE-270 is approximately 0.2–0.3 µM, and that of PFKFB3 knockout is between 0.6 and 1.0 µM, representing a nearly 3–5-fold increase (Figure 2B). For the KYSE-150 cell line, the IC50 of the WT and NC cells is around 2–5 µM, and the value after PFKFB3 knockout is around 7–10 µM, which is also a nearly 2–3-fold increase (Figure 2C). The findings indicate that the knockout of PFKFB3 resulted in increased resistance to 5-FU in these three human esophageal cancer cell lines.

### 2.3. The Impact of PFKFB3 on Apoptosis in Human Esophageal Cancer Cell Lines

In order to explore the mechanism behind the increased resistance to 5-FU after PFKFB3 knockout, we first utilized flow cytometry with Annexin V-APC/PI staining to detect the apoptosis status of esophageal cancer cells before and after 5-FU treatment. Regarding the conditions for the 5-FU treatment groups, the three cell lines were treated with different concentrations of 5-FU for 72 h: KYSE-70: 10 µM; KYSE-270: 1.5 µM; KYSE-150: 10 µM.

**Figure 3:**
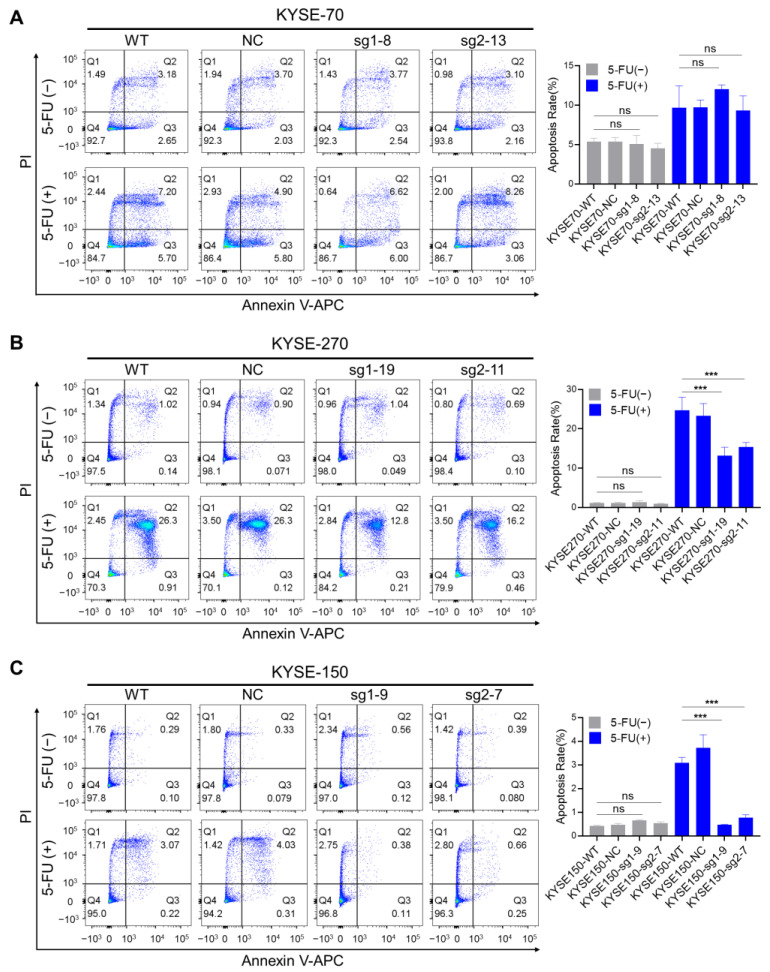
The impact of PFKFB3 on apoptosis in human esophageal cancer cell lines. (**A**–**C**) Effect of PFKFB3 in the apoptosis of esophageal cancer cells during 5-FU treatment. The apoptotic status of the cells was evaluated via Annexin-V/PI staining. Representative dot plots show four quadrants: necrotic cells (Q1, Annexin-V^−−^/PI^+^, upper left), late apoptotic cells (Q2, Annexin-V^+^/PI^+^, upper right), early apoptotic cells (Q3, Annexin-V^+^/PI^−−^, lower right), live cells (Q4, Annexin-V^−−^/PI^−−^, lower left). Percentages indicate the proportion of cells in each quadrant. The apoptosis rate was calculated as the sum of the late apoptotic (Q2) and early apoptotic (Q3) cell populations. The data are presented as the mean of three independent experiments. The statistical significance of the results was determined using one-way ANOVA, with the following *p*-values: *** *p* < 0.001.

Our results show that in the absence of 5-FU treatment, no significant changes were observed between the WT cells and the PFKFB3 knockout cells in all three esophageal cancer cell lines. However, interestingly, under 5-FU treatment (10 µM 5-FU, 72 h), no significant changes in cell apoptosis were observed in the KYSE-70 cell line (Figure 3A). However, in the KYSE-270 (1.5 µM 5-FU, 72 h) and KYSE-150 (10 µM 5-FU, 72 h) cell lines, there was a significant decrease in apoptosis rates (Q2 + Q3) in the PFKFB3 knockout group. The original data are described as follows: KYSE270-WT: 24.69 ± 3.356; KYSE270-NC: 23.34 ± 3.085; KYSE270-sg1-19: 13.13 ± 2.168; KYSE270-sg2-11: 15.33 ± 1.179 (Figure 3B). KYSE150-WT: 3.08 ± 0.239; KYSE150-NC: 3.713 ± 0.5605; KYSE150-sg1-9: 0.4733 ± 0.01528; KYSE150-sg2-7: 0.77 ± 0.1229 (Figure 3C). These preliminary results suggest that PFKFB3 knockout may contribute to increased resistance to 5-FU in the KYSE-270 and KYSE-150 cell lines by suppressing the apoptotic pathway.

### 2.4. The Impact of PFKFB3 on Cell Cycle in Human Esophageal Cancer Cell Lines

Notably, after 72 h of 5-FU treatment, the concentrations of 5-FU were as follows: KYSE-70: 10 µM; KYSE-270: 1.5 µM; KYSE-150: 10 µM. The S phase was significantly prolonged in PFKFB3-deleted cells in the KYSE-70 and KYSE-270 cell lines. The original data are described as follows: KYSE70-WT: 30.7 ± 4.757; KYSE70-NC: 17.10 ± 3.219; KYSE70-sg1-8: 40.07 ± 2.754; KYSE70-sg2-13: 49.47 ± 8.462 (Figure S3A). KYSE270-WT: 41 ± 0.2646; KYSE270-NC: 39.97 ± 1.012; KYSE270-sg1-19: 48.6 ± 3.245; KYSE270-sg2-11: 55.47 ± 1.943 (Figure S3B). However, there was no significant difference in the S phase in the KYSE-150 cell line under the 5-FU treatment.

In the absence of 5-FU, flow cytometry analysis indicated that there was no significant difference in the S phase between PFKFB3-deleted cells and WT cells in the KYSE-70 and KYSE-270 cell lines. However, in the KYSE-150 cell line, PFKFB3 deficiency resulted in an extended S phase. The original data are described as follows: KYSE150-WT: 6.623 ± 0.195; KYSE150-NC: 7.163 ± 1.873; KYSE150-sg1-9: 12.87 ± 2.139; KYSE150-sg2-7: 13.9 ± 3.35 (Figure S3C).

### 2.5. The Impact of PFKFB3 on EMT in Human Esophageal Cancer Cell Lines

**Figure 4:**
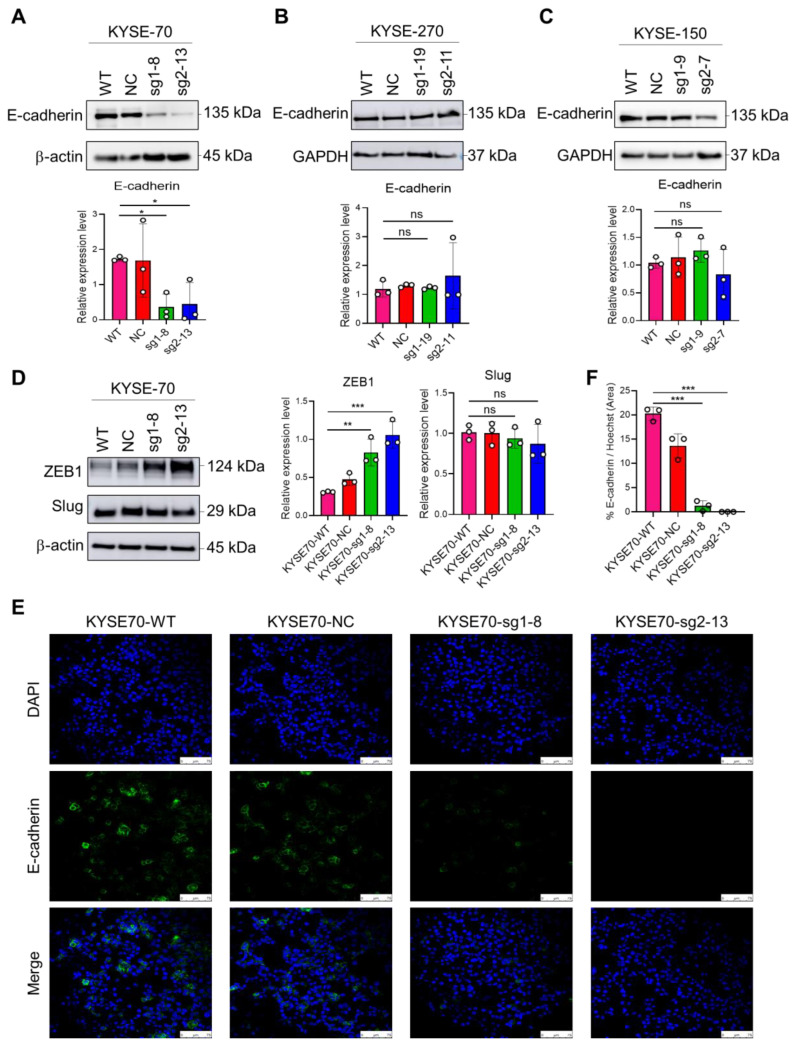
The impact of PFKFB3 on EMT in human esophageal cancer cell lines. (**A**) Western blot analysis shows significant downregulation of E-cadherin in the PFKFB3 knockout KYSE-70 cell line compared to control cells. β-actin was used as a loading control. (**B**,**C**) No significant differences in E-cadherin expression were observed between PFKFB3 knockout and control cells in both KYSE-270 and KYSE-150 cell lines. GAPDH was used as a loading control. (**D**) PFKFB3 knockout of KYSE-70 cell lines results in significant upregulation of ZEB1 expression, with no significant changes observed in Slug expression. β-actin was used as a loading control. The data are presented as the mean of three independent experiments. The statistical significance of the results was determined using one-way ANOVA, with the following *p*-values: * *p* < 0.05; ** *p* < 0.01; and *** *p* < 0.001. (**E**) Immunofluorescence staining of E-cadherin (green), DAPI (blue), and merge figure in KYSE-70 cell line. Scale bar = 75 µM. The staining shows significant reduction in E-cadherin expression in PFKFB3 knockout group compared to control group. (**F**) Quantification of E-cadherin immunofluorescence intensity, analyzed using ImageJ. Data represent statistical analysis from three independent experiments.

Western blot analysis shows the expression changes in E-cadherin, ZEB1, and Slug in the PFKFB3 knockout group compared to the control group. In the KYSE-70 cell line, E-cadherin expression is significantly downregulated in the PFKFB3 knockout group, indicating the occurrence of EMT (Figure 4A). In the KYSE-270 and KYSE-150 cell lines, E-cadherin expression shows no significant difference between the PFKFB3 knockout group and the control group (Figure 4B,C). ZEB1 expression is significantly upregulated in the PFKFB3 knockout group of the KYSE-70 cell line, suggesting its key regulatory role in the EMT process. Slug expression shows no significant changes (Figure 4D). The expression levels of each protein were normalized to β-actin or GAPDH as a loading control for comparison. The intensity of the Western blot bands was quantified using Image J analysis software (version 1.53t; National Institutes of Health, Bethesda, MD, USA; RRID:SCR_003070). The grayscale values for each band were measured, and background subtraction was performed to obtain corrected intensities. The data were then normalized to the loading control and expressed as relative intensity compared to the control group.

In the KYSE-70 cell line, immunofluorescence staining revealed a significant reduction in E-cadherin (green) on the cell membrane in the PFKFB3 knockout group (Figure 4E,F). This observation indicates a loss of cell–cell adhesion, thereby supporting the occurrence of EMT. This observed change through immunofluorescence microscopy is consistent with our Western blot results, where we similarly observed a decrease in E-cadherin expression in the PFKFB3 knockout group.

### 2.6. Loss of PFKFB3 Indirectly Regulates Phosphorylation of Checkpoint Kinase (Chk1) by Increasing the Expression of Slug and Mcl-1, Thereby Inhibiting 5-FU-Induced Apoptosis in KYSE-270 and KYSE-150 Cells

**Figure 5:**
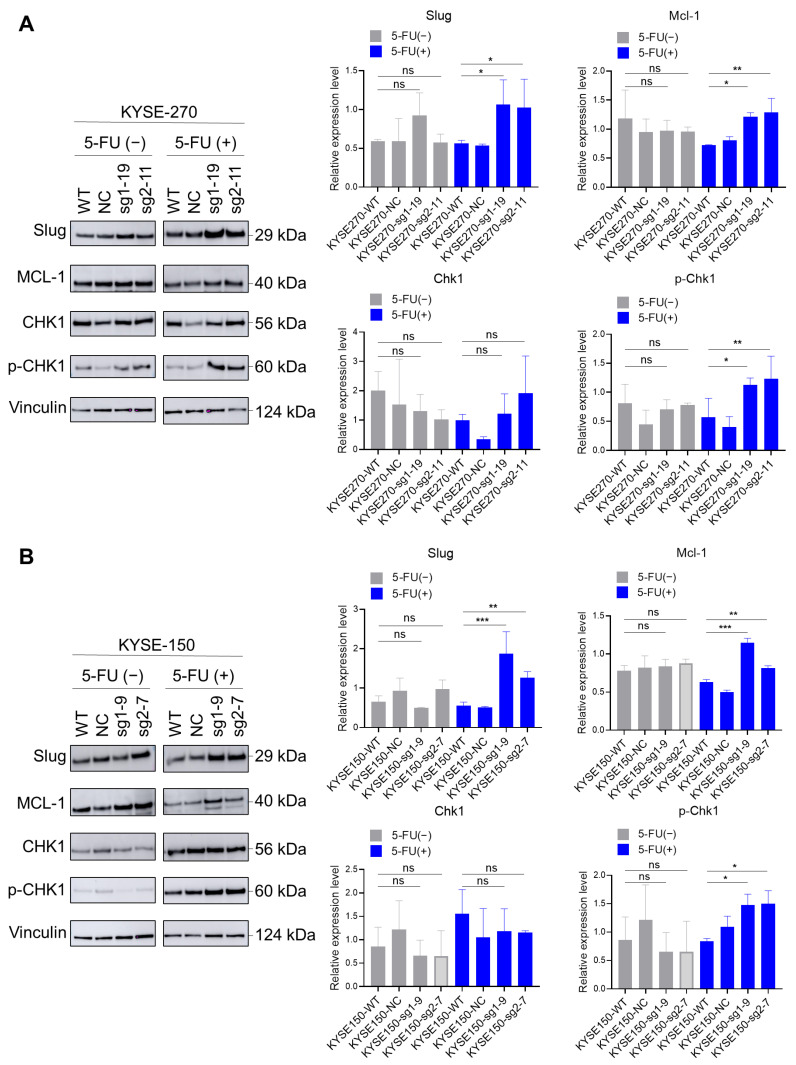
Loss of PFKFB3 indirectly regulates phosphorylation of checkpoint kinase (Chk1) by increasing the expression of Slug and Mcl-1, thereby inhibiting 5-FU-induced apoptosis in KYSE-270 and KYSE-150 cells. Western blot analysis of Slug, MCL-1, Chk1 and p-Chk1 expression before and after 5-FU treatment in KYSE-270 (**A**) and KYSE-150 (**B**) cell lines. Vinculin was used as a loading control. The data are presented as the mean of three independent experiments. The statistical significance of the results was determined using one-way ANOVA, with the following *p*-values: * *p* < 0.05; ** *p* < 0.01; and *** *p* < 0.001.

In our experiments, we designed the no 5-FU group and the 5-FU group to further clarify the mechanism between PFKFB3 deletion and 5-FU resistance in the KYSE-270 (Figure 5A) and KYSE-150 (Figure 5B) cell lines. Regarding the conditions for the 5-FU treatment groups, these two cell lines were treated with different concentrations of 5-FU for 72 h: KYSE-270: 1.5 µM; KYSE-150: 10 µM.

In the absence of 5-FU, we observed no significant changes in the expression of Slug, Mcl-1, Chk1, and p-Chk1 in the PFKFB3 knockout group compared to the control group. However, when 5-FU was applied, we found that the expression of Slug and Mcl-1 was significantly upregulated in PFKFB3-deleted cells compared to controls. Mcl-1 is an anti-apoptotic protein known to promote cell survival and inhibit apoptosis. In contrast, Slug is an EMT transcription factor closely associated with apoptosis.

Furthermore, we observed that PFKFB3 deletion affects the phosphorylation status of checkpoint kinase 1 (Chk1), a key regulator of cell cycle checkpoints and DNA damage response pathways. In our experiments, we found that the phosphorylation level of Chk1 was significantly increased in PFKFB3-deleted cells, suggesting an abnormality in the DNA damage response pathway.

Overall, the loss of PFKFB3, upregulation of Slug and Mcl-1, and increased phosphorylation levels of Chk1 collectively resulted in significant inhibition of apoptosis in KYSE-270 and KYSE-150 cells induced via 5-FU. This indicates that the loss of PFKFB3 confers resistance to 5-FU-induced apoptosis in these cell lines, possibly through the modulation of key signaling pathways involving Slug, Mcl-1, and Chk1.

## 3. Discussion

The role of cancer gene mutations in individualized precision therapy is crucial. Individualized precision therapy is a treatment tailored to each patient’s specific genes, environment, and lifestyle. Certain mutations can make cancer cells particularly sensitive to specific drugs or lead to drug resistance [19,20,21]. In cancer treatment, mutation analysis can guide the choice of targeted therapy [22,23,24]. Our study, therefore, used CRISPR/Cas9 screening technology to identify key genes associated with 5-FU resistance in esophageal cancer from a library of more than 1000 human protein kinases. The positive selection results of CRISPR/Cas9 screening identified several kinases. One of these four genes, Uridine-cytidine-kinase (UCK2), also known as Uridine monophosphate kinase (UMPK), is a key protein kinase in 5-FU metabolism, and many studies have shown decreased levels of UMPK as a mechanism of resistance to 5-FU [25,26]. This also indirectly proves the reliability and credibility of our screening data. Interestingly, our screening results identified another novel candidate gene, PFKFB3, suggesting that it may play a role in the process of 5-FU resistance. Our study demonstrates that the loss of PFKFB3 increased 5-FU resistance in esophageal cancer cell lines, which we successfully validated in three human esophageal cancer cell lines: KYSE-70, KYSE-270, and KYSE-150.

PFKFB3 (6-phosphofructo-2-kinase/fructose-2,6-biphosphatase3) is mapped to a single locus on chromosome 10 (10p15-p14) [27]. It is one of four PFKFB isozymes and a key enzyme responsible for controlling glycolysis [27]. The PFKFB family of enzymes are essential glycolytic activators whose function is to catalyze the synthesis and degradation of fructose-2,6-bisphosphate (F2,6BP)—an allosteric activator of 6-phosphofructo-1-kinase (PFK-1), a rate-limiting enzyme and essential control point in the glycolytic pathway [28,29]. Under normal physiological conditions, the PFKFB3 gene is expressed at relatively low levels in a variety of human tissues but is overexpressed in a number of malignant tumors. As a key regulator of the glycolysis pathway, PFKFB3 has become a focus of research in a variety of cancer cells [30,31,32,33]. Previous studies have focused on exploring the relationship between PFKFB3 and cancer cell growth, proliferation, migration and metastasis [30,31,32,33], but relatively few studies have investigated the relationship between PFKFB3 and cancer drug resistance. In our study, we investigated the potential role of PFKFB3 in 5-FU resistance in human esophageal squamous cell carcinoma and revealed its possible impact on the response of cancer cells to treatment.

So why is there an increase in 5-FU resistance in cancer cells after PFKFB3 knockout in esophageal cancer cell lines? Fabrizio M et al. summarized and described four basic mechanisms through which glycolysis induces drug resistance [34]: 1. inhibition of apoptosis; 2. induction of epithelial–mesenchymal transition; 3. induction of autophagy; 4. inhibition of drug influx and enhancement of drug efflux. PFKFB3 has also been linked to apoptosis and cell cycle progression in several studies. Apoptosis is the main mechanism through which cancer cells die in response to anti-cancer drugs; therefore, molecules that induce drug resistance are expected to have anti-apoptotic effects.

Our apoptosis results show that under normal conditions (no 5-FU group), apoptosis did not change significantly before and after PFKFB3 knockout. In contrast, apoptosis was significantly reduced after PFKFB3 knockout in the KYSE-270 and KYSE-150 cell lines when 5-FU was applied; interestingly, no significant apoptotic changes were observed in the KYSE-70 cell line. The results of this study tentatively suggest that there may be different mechanisms behind the development of 5-FU resistance against three different esophageal cancer cell lines. This suggests that different cell lines may resist the therapeutic effects of 5-FU through different pathways, demonstrating the diversity and complexity of drug resistance in cancer.

As is well known, treating cells with 5-FU can lead to DNA damage, particularly during the S phase, where erroneous incorporation of FdUTP into DNA causes double-strand (and single-strand) breaks [5,35]. However, DNA damage can occur at all stages of the cell cycle in proliferating cells, and the repair mechanisms involved vary at different stages of the cell cycle. Therefore, in subsequent experiments, we used flow cytometry to observe changes in the cell cycle before and after PFKFB3 knockout and before and after 5-FU treatment, particularly focusing on changes during the S phase. Our cell cycle results indicate that the effect of PFKFB3 knockout on the S phase of the cell cycle varies among the three esophageal cancer cell lines. In the absence of 5-FU treatment, there were no significant changes observed in the PFKFB3 knockout groups compared to the control groups in the KYSE-70 and KYSE-270 cell lines. However, in the KYSE-150 cell line, PFKFB3 knockout led to an extension of the S phase. Interestingly, when treated with 5-FU, we observed a significant extension of the S phase in the PFKFB3 knockout groups compared to the control groups in the KYSE-70 and KYSE-270 cell lines. There was no significant difference in the S phase of the KYSE-150 cell line. This result once again underscores the presence of distinct biological characteristics among different cell lines.

After 5-FU induces DNA damage, the body mitigates the damage and ensures genomic stability through the DNA damage response (DDR) [36]. Checkpoint kinases are protein kinases involved in cell cycle control, and two types of checkpoint kinase subtypes have been identified: Chk1 and Chk2 [37]. Chk1 is a crucial factor in cell cycle checkpoint regulation and a major regulator of DDR. It activates checkpoints, delays cell cycle progression, and provides time for DNA repair, which is essential for maintaining genomic integrity [38]. For instance, the S phase checkpoint slows down DNA synthesis during replication stress, ensuring the accurate replication of DNA-damaged S-phase cells [38]. According to multiple studies, Chk1 is phosphorylated at several sites in response to DNA damage and participates in DNA damage repair in its phosphorylated form [37,38]. This includes ATR-mediated phosphorylation at Ser317 and Ser345, as well as autophosphorylation at Ser296 [39]. We also examined the expression of Chk1 and p-Chk1 through Western blot analysis. The results showed that regardless of the 5-FU treatment, PFKFB3 knockout did not affect Chk1 expression. However, in the KYSE-270 and KYSE-150 cell lines, p-Chk1(Ser296) significantly increased in the PFKFB3 knockout group upon 5-FU exposure. This may imply that the absence of PFKFB3 induces Chk1 autophosphorylation in response to 5-FU-induced DNA damage, thereby prolonging the S phase and allowing more time for DNA damage repair. This could lead to greater tumor cell survival or apoptosis inhibition. This might also explain why PFKFB3 knockout in these two cell lines suppresses apoptosis. To verify this hypothesis, we further examined the expression of Mcl-1.

Mcl-1 is a multifunctional regulatory protein and the first anti-apoptotic member identified in the Bcl-2 family [40]. It is involved in apoptosis, differentiation, and cell cycle regulation of various cancer cells and has a close relationship with chemotherapeutic agents [40]. Several studies have shown that the overexpression of the Bcl-2 family allows cancer cells to evade apoptosis, providing a survival advantage and contributing to the development of drug resistance in tumor cells [41]. Our Western blot results also indicate that Mcl-1 expression in the KYSE-270 and KYSE-150 cell lines increases in the PFKFB3 knockout group under the 5-FU treatment. This finding is consistent with the flow cytometry apoptosis results. Several studies have also revealed a relationship between Mcl-1 and Chk1, suggesting that Chk1 phosphorylation is indirectly regulated by Mcl-1 [42]. Our experimental results align with this, indicating that PFKFB3 knockout upregulates the expression of the anti-apoptotic protein Mcl-1 and promotes the expression of phosphorylated checkpoint kinase 1 (p-Chk1). This regulation of the cell cycle and inhibition of apoptosis allows tumor cells to evade the effects of 5-FU.

In our previous discussion, we mentioned that glycolytic metabolism can induce EMT and lead to drug resistance [34]. Other studies have also shown a close relationship between EMT and 5-FU resistance in various tumors [43,44,45,46]. To further explore the underlying mechanisms of 5-FU resistance in these three esophageal cancer cell lines, we examined the changes in EMT-related proteins, such as E-cadherin, and EMT transcription factors, including Slug and ZEB1. Our results show that in the KYSE-70 cell line, the expression of ZEB1 was significantly increased in the PFKFB3 knockout group compared to the WT group, while the expression of E-cadherin was reduced. These results suggest that loss of PFKFB3 may induce EMT and contribute to 5-FU resistance in the KYSE-70 cell line.

In the KYSE-270 and KYSE-150 cell lines, loss of PFKFB3 did not result in significant changes in the expression of E-cadherin and Slug, suggesting that EMT is not essential for 5-FU resistance in these two cell lines. However, with 5-FU treatment, Slug expression was significantly increased in PFKFB3-deleted cells compared to WT cells in both cell lines. The role of these transcription factors in EMT has been validated and recognized many times. Since Slug and ZEB1 have different expression patterns, their contributions to EMT are thought to depend on the specific cell or tissue type and the signaling pathways involved [47]. Slug, a member of the Snail family, is a highly conserved zinc transcription factor [48,49]. In addition to its role in EMT, Slug was shown to inhibit apoptosis in cancer and other cells by transactivating the expression of PUMA, Bcl-2 and Bax, thereby exerting an anti-apoptotic effect [50,51]. Slug was also shown to promote cell survival by blocking DNA damage-induced apoptosis [49]. This helps to explain why, in our results, Slug expression in the PFKFB3 knockout group increased only under 5-FU treatment. This suggests that loss of PFKFB3 leads to increased Slug expression upon 5-FU treatment, which subsequently regulates the activation of Mcl-1 and exerts an anti-apoptotic effect, thereby contributing to 5-FU resistance.

**Figure 6:**
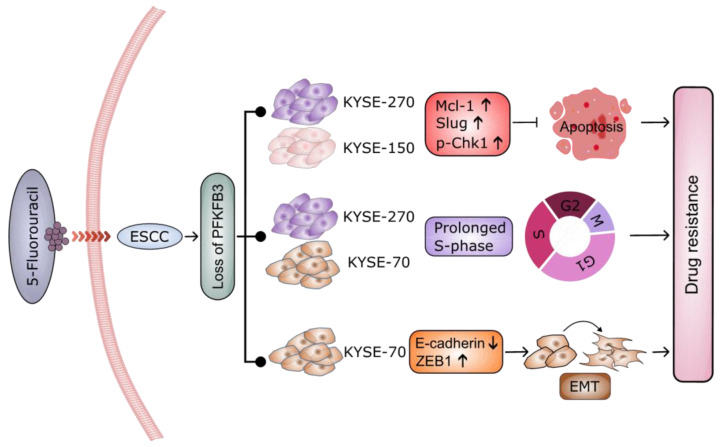
Schematic of the research outcome. Loss of PFKFB3 increases the resistance of KYSE-70, KYSE-270, and KYSE-150 esophageal squamous cell carcinoma cells to 5-FU. Specifically, in KYSE-70 cells, loss of PFKFB3 can induce EMT and prolong the S phase of the cell cycle, allowing cancer cells to evade the effects of 5-FU and develop resistance. In KYSE-270 and KYSE-150 cell lines, loss of PFKFB3 can upregulate the expression of Slug and Mcl-1, indirectly regulate Chk1, and promote its autophosphorylation, which in turn inhibits apoptosis, thus counteracting the effects of 5-FU.

In summary, we can be certain that the loss of PFKFB3 increases the resistance of KYSE-70, KYSE-270 and KYSE-150 esophageal squamous cell carcinoma cells to 5-FU (Figure 6). Specifically, in KYSE-70 cells, loss of PFKFB3 can induce EMT and prolong the S phase of the cell cycle, allowing cancer cells to evade the effects of 5-FU and develop resistance. In KYSE-270 and KYSE-150 cell lines, loss of PFKFB3 can upregulate the expression of Slug and Mcl-1, indirectly regulate Chk1, and promote its autophosphorylation, which in turn inhibits apoptosis, thus counteracting the effects of 5-FU.

The results of this study preliminarily suggest that different mechanisms may underlie the development of 5-FU resistance in three different esophageal cancer cell lines. This implies that different cell lines may resist 5-FU treatment through different pathways, highlighting the diversity and complexity of cancer cell resistance. However, traditional experimental methods have limitations in fully explaining the complex resistance mechanisms of different cell lines during chemotherapy. Resistance is often related to protein mutations, structural changes, and dysfunctions in tumor cells, but these changes are difficult to observe directly.

In recent years, AlphaFold, developed by DeepMind, has shown great value in structural biology and drug discovery by accurately predicting protein 3D structures based on amino acid sequences [52,53,54,55]. AlphaFold can help researchers identify structural changes in proteins caused by mutations, which may be key factors in drug resistance. For example, AlphaFold was successfully applied in drug development for hepatocellular carcinoma, where predicted protein structures were used to design potential therapeutic compounds in a short period of time [56]. This provides a new perspective for studying the mechanisms of chemotherapy resistance in cancer.

In the future, the use of AlphaFold in research will help us gain a clearer understanding of resistance mechanisms in different tumor cell lines, providing useful insights for creating new strategies to overcome resistance. By combining traditional methods with the advanced technology of AlphaFold, we can speed up progress in cancer resistance research and create new possibilities for precision and personalized cancer treatments.

## 4. Conclusions

In conclusion, our research findings indicate that the loss of PFKFB3 can increase the resistance of different human esophageal squamous cell carcinoma cell lines to 5-FU through various pathways. This not only enriches our understanding of the biological characteristics of different ESCC cell lines but also provides new clinical insights for future personalized treatment. For ESCC patients with different genetic backgrounds, assessing the status of PFKFB3 could help predict their sensitivity or resistance to 5-FU, allowing for more precise treatment plans. Such personalized treatment strategies have the potential to improve treatment efficacy, reduce unnecessary drug use and side effects, and ultimately enhance patient survival rates and quality of life.

## 5. Methods

### 5.1. Cell Culture

Human esophageal cancer KYSE-70 (DSMZ, NO. ACC 363, RRID:CVCL_1356), KYSE-270 (DSMZ, NO. ACC 380, RRID:CVCL_1350), KYSE-150 (DSMZ, NO. ACC 375, RRID:CVCL_1348) cell lines were purchased from DSMZ-German Collection of Microorganisms and Cell Cultures GmbH. KYSE-70 was cultured in 90% RPMI (Roswell Park Memorial Institute) 1640 medium (Thermo Fisher Scientific, Darmstadt, Germany, #31870-025) with 10% heat-inactivated (56 °C for 30 min) fetal bovine serum (Thermo Fisher Scientific, Germany, #A3160801). KYSE-270 and KYSE-150 were cultured in 49% RPMI 1640 medium and 49% DMEM/F12 (Dulbecco’s Modified Eagle’s Medium/Nutrient Mixture F-12) (Thermo Fisher Scientific, Germany, #11320-074) medium with 2% heat-inactivated FBS. HEK293TN (BioCat, LV900A-1-GVO-SBI, RRID:CVCL_UL49) was cultured in 90% DMEM (Dulbecco’s Modified Eagle’s Medium) (Thermo Fisher Scientific, Germany, #31966-021) supplemented with 10% heat-inactivated FBS. Cell cultures were incubated in a humidified atmosphere of 95% air and 5% CO2 at a constant temperature of 37 °C. DPBS (Dulbecco’s phosphate-buffered saline) (Thermo Fisher Scientific, Germany, #14190094) was used to wash the cells, and all cells were harvested with 0.25% Trypsin-Ethylenediaminetetraacetic acid (EDTA) (Thermo Fisher Scientific, Germany, #25200-072).

### 5.2. Production of Pooled sgRNA Library Lentivirus

The human protein kinase library, which was a gift from John Doench and David Root (RRID: Addgene_75312), and additional sgRNAs designed to target genes involved in protein kinase [57] contained 6934 sgRNAs targeting 1053 human genes and 107 NTCs (non-targeting-controls). Approximately 18–24 h before transfection, a T75 flask was seeded with ~7 × 10^6^ HEK293TN cells in a total volume of 12 mL of D10 medium (90% DMEM + 10% heat-inactivated FBS), incubated overnight. After the cells reached 95–99% confluency, lentivirus plasmid transfection was carried out. Lentivirus production requires the co-transfection of accessory helper plasmids that encode the necessary genes for packaging and generating lentiviral particles. We used 2.8 µg pMDLg/pRRE plasmid (Addgene plasmid #12,251;; RRID:Addgene_12251), 1.4 μg pRSV-REV plasmid (Addgene plasmid #12,253; RRID:Addgene_12253), 1.4 μg pMD2.G plasmid (Addgene plasmid #12,259; http://n2t.net/addgene:12259; RRID:Addgene_12259), 4.3 µg pooled-sgRNA library plasmid, 19.8 µL P3000 Enhancer Reagent (Invitrogen, Thermo Fisher Scientific, Germany, #L3000015) and 41 µL Lipofectamine 3000 transfection reagent (Invitrogen, Thermo Fisher Scientific, Germany, #L3000015) mixed in Opti-MEM I reduce serum medium (Thermo Fisher Scientific, Germany, #31985070) and added into the HEK293TN cells. At 6 h post-transfection, 12 mL of pre-warmed D10 medium was replaced and incubated overnight. After 24 h, the virus was collected using a 0.45 µm pore filter. The pMDLg/pRRE, pRSV-REV, and pMD2.G were gifts from Didier Trono.

### 5.3. Lenti-Library-Puro Transduction

Before starting, the antibiotic concentration was determined for selection in our cell line. In this study, we used 5 µg/mL puromycin (InvivoGen, USA, #ant-pr-1) for three days to select KYSE-70 and KYSE-270 cells transduced with the lenti-library. The transduction conditions were as follows: 1.8 × 10^6^ cells/T75 flask, 4 µg/mL polybrene, 12 mL medium and different lentiviral supernatant volumes. We normally perform transductions for CRISPR-suppressor scanning experiments as a multiplicity of infection (MOI) < 0.3. Each library cell was standardized for >500× coverage of the library in order to ensure sufficient representation of all sgRNAs.

### 5.4. CRISPR/Cas9 Screening with 5-FU

Both KYSE-70 and KYSE-270 cells were transduced in triplicate with the lenti-library, and the remaining surviving cells were continuously cultured and divided into two groups. With 5-FU as a treatment group, the screening conditions were as follows: 5.47 × 10^6^ cells/T175 flask, 35 mL total volume, KYSE-70 (IC90 = 20 µM 5-FU) and KYSE-270 (IC90 = 1.5 µM 5-FU) for 3 weeks, and replace the medium with 5-FU every 3 days. While untreated cells were continuously cultured for 3 weeks as a control group, it was ensured that at least 10 million cells were collected from each group for the following deep sequencing. 5-FU (50 mg/mL) was purchased from the pharmacy of the Universitätsklinikum Erlangen in ready-made solutions.

### 5.5. Isolation of Genomic DNA and Deep Sequencing

Genomic DNA was extracted from the cell pellets using the NucleoSpin^®^ Blood L (Machery Nagel, Dueren, Germany, #740954.20) according to the manufacturer’s instructions. Extracted gDNA was amplified with the following parameters: each 100 µL reaction can accommodate up to 10 µg of gDNA template, 50 µL Q5^®^ Hot Start High-Fidelity 2x Master Mix (New England Biolabs, Frankfurt, Germany, #M0494L), 3 µL P5 (10pmol) primer, 3 µL P7(10 pmol) primer, and ddH2O. To obtain 500× coverage, 20 µg of gDNA was utilized for each sample. The completed reactions were run on a 1% agarose gel in TAE buffer to confirm the presence of the expected PCR products at around 370 bp. The DNA concentration of the PCR product was quantified using a Qubit dsDNA-High Sensitivity assay kit (Thermo Fisher Scientific, Germany, #Q32850) according to the manufacturer’s protocol. The samples were submitted for next-generation sequencing (NGS), performed on the Illumina HiSeq 2500 platform (Illumina, San Diego, CA, USA) in the deep sequencing facility of TU Dresden. The deep sequencing data were evaluated and analyzed with the MAGeCK-VISPR program. P5 and P7 primers were used and synthesized by Eurofins Genomics (Table 2).

### 5.6. Detection of Cell Viability

In total, 1 × 10^4^ cells per well were seeded into a 96-well plate (Corning, NY, USA, #3603). The drug treatment was administered after the cells adhered overnight. After 3 days of drug treatment, the cells were stained with DAPI and Hoechst dyes (Life Technologies, Darmstadt, Germany, #H3570), and imaged in 9 fields for each well using the Evos FL Auto 2 imaging system (Invitrogen, Bothell, WA, USA, #AMAFD2000). The images were analyzed using HCS studio cell analysis software V2.0 (Thermo Fisher Scientific, Waltham, MA, USA, #SX000041A). The IC50 was calculated using GraphPad Prism (RRID:SCR_002798). For KYSE-270 cells, the concentration of 5-FU ranged from 0.01 to 64 µM with 4-fold increments. For KYSE-70 and KYSE-150 cells, the concentration ranged from 0.01 to 640 µM with 4-fold increments.

### 5.7. Construction of Stable Knockout Cell Lines with CRISPR/Cas9

In this project, PFKFB3 as a target gene was knocked out using CRISPR-Cas9 system technology in three human esophageal cancer cell lines (KYSE-70, KYSE-150 and KYSE-270). We applied the experimental method from Zhang’s lab [12]. The sgRNA sequences used in this study were derived from the human protein kinase library, which was originally designed based on the optimized sgRNA selection criteria described by Doench et al. [57], ensuring high on-target activity and minimal off-target effects, and the sgRNAs were synthesized by Eurofins Genomics. First, sgRNA was cloned into the pSpCas9(BB)-2A-Puro (PX459) V2.0 vector (Addgene plasmid #62988; RRID: Addgene_62988) for co-expression with Cas9. This plasmid vector was a gift from Feng Zhang [12]. The ligation plasmid was then transformed into the Endura DUOs Electrocompetent Cells (Biosearch Technologies, Middleton, WI, USA, #60242-2) according to the protocol supplied with the cells. The plasmid DNA was isolated from single colony cultures using a GeneJET Plasmid miniprep Kit (Thermo Fisher Scientific, Germany, #K0503), and the sequences were validated via Eurofins sequencing. The CRISPR plasmid sequence validation method described in Zhang’s article was employed [12]. The sequence of each colony was verified through sequencing from the U6 promoter using the U6-Fwd primer. We transfected the pSpCas9 (BB)-2A-Puro-PFKFB3 plasmid to the cells using a lipofectamine 3000 transfection kit (Invitrogen, Thermo Fisher Scientific, Germany, #L3000015). Successfully transfected cells were seeded into a 96-well plate to collect single clones.

Monoclonal cells were obtained using the limited dilution method, in which each well ideally contains a single cell. Specifically, transfected cells were counted and diluted to a concentration of 80 cells per 12 mL of culture medium, and then seeded into 96-well plates. After 5–10 days of incubation, individual wells were examined under a microscope to identify and select wells with single-cell–derived clones. Single clone cells were verified via Western blot and Eurofins DNA sequencing (Sanger sequencing). We named the single clone cells that were finally generated and used in this study as follows: WT (wild-type cells), NC (transfected with non-targeting control sgRNA), sg1 (transfected with PFKFB3-sgRNA1), and sg2 (transfected with PFKFB3-sgRNA2).

We purchased and synthesized sgRNAs from Eurofins Genomics (Ebersberg, Germany) (Table 3).

### 5.8. Western Blot

Cells were collected and added to RIPA buffer (Thermo Fisher Scientific, Rockford, IL, USA, #89900) plus 100:1 protease and phosphatase inhibitor (Thermo Fisher Scientific, Rockford, IL, USA, #78442), and a bicinchoninic acid (BCA) protein concentration kit (Thermo Fisher Scientific, Rockford, IL, USA, #23250) was used to determine the protein concentrations. Equal amounts of protein samples were subjected to 4–12% NUPAGE Bis-Tris gels (Thermo Fisher Scientific, USA, #NP0322BOX), transferred to a nitrocellulose filter membranes (Sigma-aldrich, St. Louis, MO, USA, #GE10600003), and blocked using 5% skim milk powder or 5% BSA. Then, the following primary antibodies were incubated overnight at 4 °C: E-Cadherin (24E10) Rabbit mAb (Cell Signaling Technology, Danvers, MA, USA, Cat#3195, RRID:AB_2291471); ZAB1 (Sigma-Aldrich, St. Louis, MO, USA, Cat#HPA027524, RRID:AB_1844977); PFKFB3 (EPR12594) (Abcam, Cambridge, UK, Cat#ab181861, RRID:AB_3095816); Chk1 (Cell Signaling Technology Cat#2360, RRID:AB_2080320); phospho-Chk1 (S296) (Cell Signaling Technology Cat#90178, RRID:AB_2800153); Slug (Cell Signaling Technology Cat#9585, RRID:AB_2239535); MCL-1 (Cell Signaling Technology Cat#5453, RRID:AB_10694494). GAPDH (Cell Signaling Technology Cat#5174, RRID: AB_10622025), β-actin (Cell Signaling Technology Cat#8457, RRID: AB_10950489), and Vinculin (Cell Signaling Technology Cat#13901, RRID: AB_2728768) served as the loading controls. The membranes were washed again and incubated with secondary antibody anti-rabbit IgG, HRP-linked (Cell Signaling Technology Cat#7074, RRID: AB_2099233), or anti-mouse IgG, HRP-linked (Cell Signaling Technology Cat#7076, RRID: AB_330924) for 1 h at room temperature. The membranes were detected using an GE Amersham Imager 600 (RRID:SCR_021853) with SignalFire Elite ECL Reagent (Cell Signaling Technology Cat#12757S) or SignalFire ECL Reagent (Cell Signaling Technology Cat#6883S). Finally, the intensity of the Western blot band was quantified using ImageJ (RRID:SCR_003070).

### 5.9. Eurofins Genomics DNA Sequencing

The genomic DNA was extracted from the cell pellets using the NucleoSpin^®^ Blood L (Machery Nagel, Germany, #740954.20) according to the manufacturer’s instructions. The primers for sgRNAs targeting regions and Q5^®^ Hot Start High-Fidelity 2x Master Mix (New England Biolabs, Frankfurt, Germany, #M0494L) were used for the amplifications. PCR products were purified with Wizard^®^ SV Gel and a PCR Clean-Up System (Promega, Madison, WI, USA, #A9281) and transformed into a pMiniT 2.0 vector using an NEB PCR Cloning Kit (New England Biolabs, Germany, #E1203S). The plasmid DNA was isolated from single colony cultures using a GeneJET Plasmid miniprep Kit (Thermo Fisher Scientific, Germany, #K0503), and the plasmids were then sent to Eurofins Genomics (Ebersberg, Germany), where also synthesized the sequencing PCR primers (Table 2).

### 5.10. Apoptosis Assay

The cells were harvested with Trypsin-EDTA and washed with ice-cold (2–8 °C) PBS and resuspended in 200 µL 1 × binding buffer (BD Pharmingen, San Diego, CA, USA, #556454). The cells were subsequently incubated with 5 µL Annexin V-APC (BD Pharmingen, San Diego, CA, USA, #550474) and 5 µL Propidium Iodide (PI) Solution (Biolegend, San Diego, CA, USA, #421301) at room temperature for 15 min in the dark. All samples were detected using a BD LSR II Flow Cytometer (BD Biosciences, San Jose, CA, USA, RRID:SCR_002159). The apoptosis (early + late) results were quantified and analyzed using the FlowJo v10.8 Software (RRID:SCR_008520).

### 5.11. Cell Cycle Analysis

The cells were collected and fixed with pre-cold 70% ethanol at −20 °C overnight. The cell pellet was centrifuged and washed with ice-cold PBS two times, and then resuspended in Propidium Iodide Solution (Biolegend, San Diego, CA, USA, #421301) and RNase A (Machery Nagel, Germany, #R2045S) for 15 min at room temperature. All samples were detected using a BD LSR II Flow Cytometer (BD Biosciences, San Jose, CA, USA, RRID:SCR_002159). The results were quantified and analyzed using the FlowJo v10.8 Software (RRID:SCR_008520).

### 5.12. Immunofluorescence Staining

In total, 3 × 10^5^ cells/well were uniformly seeded onto four-well Chambered cell culture slides (SPL Life Sciences, Pocheon, Korea, #30104). After the cells were evenly distributed and growing, they were fixed with 4% Formaldehyde solution at room temperature for 15 min; rinsed with TBS three times, 5 min each; permeabilized with 0.1% Triton X-100 at room temperature for 30 min; and then rinsed with TBS twice, 5 min each. A total of 10% goat normal serum (GNS) was added, and the cells were then incubated at room temperature for 10 min to block non-specific antigens. E-cadherin antibody (Cell Signaling Technology, Danvers, MA, USA, Cat#3195, RRID:AB_2291471) diluted at 1:100 in 5% GNS was added and then incubated at 4 °C overnight. The next day, they were rinsed with TBS twice, 5 min each; then incubated with a fluorescent secondary antibody (Alexa Fluor 488 goat anti-rabbit IgG (Thermo Fisher Scientific Cat#A-11034, RRID: AB_2576217)) at room temperature for 1 h; rinsed with TBS twice, 5 min each; stained with DAPI (Thermo Fisher Scientific, MD, USA, #62248) at room temperature for 10 min; rinsed with TBS twice, 5 min each. Images were captured using a DMI 6000 CS (Leica Microsystems, Wetzlar, Germany) confocal microscope.

### 5.13. Statistical Analysis

The data were analyzed using GraphPad Prism 10.0 (RRID:SCR_002798) and expressed as the mean ± standard deviation. One-way analysis of variance (ANOVA) was used to compare the means of multiple groups. The graphs present the *p*-values: * *p* < 0.05; ** *p* < 0.01; and *** *p* < 0.001. ns means not significant. In all analyses, the results were considered statistically significant at *p* < 0.05.

## Figures and Tables

**Table 1:** Eurofins genomic sequencing confirmation of single clone cells.

KO	Cells	Mutation Number	Mutation Type	Mutation Size
A.	KYSE-70-sg1-8	1	Insertion	2 bp
B.	KYSE-70-sg2-13	1	Insertion	1 bp
C.	KYSE-150-sg1-9	2	Deletion Deletion	43 bp 124 bp
D.	KYSE-150-sg2-7	1	Insertion	1 bp
E.	KYSE-270-sg1-19	1	Deletion	5 bp
F.	KYSE-270-sg2-11		Insertion	1 bp
		2	Deletion	1 bp

**Table 2:** List of primers.

Primer	Sequence (5′-3′)
Hu_PFKFB3_sg1_forward	CCAGCGCTAAGCAGTGTAGA
Hu_PFKFB3_sg1_reverse	CCAGTGGTCCTGTGGGTAAC
Hu_PFKFB3_sg2_forward	GAGCAAGTTCGTGGAGGAGCAGA
Hu_PFKFB3_sg2_reverse	TCCCCGGTGGGGTAGCGGTAATA
P5	ACACTCTTTCCCTACACGACGCTCTTCCGAT CTNNNNNTCTTGTGGAAAGGACGAAACACCG
P7	TCTACTATTCTTTCCCCTGCACTGT

**Table 3:** List of sgRNAs.

sgRNA	Sequence (5′-3′)
Hu_PFKFB3_sg1_forward	CACCgGACATCTCTCAAGGCAGCTA
Hu_PFKFB3_sg1_reverse	AAACTAGCTGCCTTGAGAGATGTCC
Hu_PFKFB3_sg2_forward	CACCgGGCGCTCAATGAGATCGACG
Hu_PFKFB3_sg2_reverse	AAACCGTCGATCTCATTGAGCGCCC
Hu_None-targeting control_forward	CACCgGAAATGCTATGCTTCGGTTC
Hu_None-targeting control_reverse	AAACGAACCGAAGCATAGCATTTCC

## Data Availability

The data presented in this study are available in the article and in the Supplementary Materials.

## Supplementary Materials

The following supporting information can be downloaded at: https://www.mdpi.com/article/10.3390/cancers17101637/s1, Figure S1: The quality control (QC) view of MAGeCK-VISPR; Figure S2: Sanger sequencing validation of mutations in KYSE-70, KYSE-270 and KYSE-150 cell lines; Figure S3: The impact of the PFKFB3 on cell cycle in human esophageal cancer cell lines; Table S1: List of CRISPR-Cas9 screening results; Supplementary File S1: Western Blot raw data.

## References

[1] Obermannova R., Alsina M., Cervantes A., Leong T., Lordick F., Nilsson M., van Grieken N.C.T., Vogel A., Smyth E.C. (2022). Oesophageal cancer: ESMO Clinical Practice Guideline for diagnosis, treatment and follow-up. Ann. Oncol..

[2] Liu C.Q., Ma Y.L., Qin Q., Wang P.H., Luo Y., Xu P.F., Cui Y. (2023). Epidemiology of esophageal cancer in 2020 and projections to 2030 and 2040. Thorac. Cancer.

[3] Lohan-Codeco M., Barambo-Wagner M.L., Nasciutti L.E., Ribeiro Pinto L.F., Meireles Da Costa N., Palumbo A. (2022). Molecular mechanisms associated with chemoresistance in esophageal cancer. Cell. Mol. Life Sci..

[4] Deboever N., Jones C.M., Yamashita K., Ajani J.A., Hofstetter W.L. (2024). Advances in diagnosis and management of cancer of the esophagus. BMJ.

[5] Ghafouri-Fard S., Abak A., Tondro Anamag F., Shoorei H., Fattahi F., Javadinia S.A., Basiri A., Taheri M. (2021). 5-Fluorouracil: A Narrative Review on the Role of Regulatory Mechanisms in Driving Resistance to This Chemotherapeutic Agent. Front. Oncol..

[6] Sun J.M., Shen L., Shah M.A., Enzinger P., Adenis A., Doi T., Kojima T., Metges J.P., Li Z., Kim S.B. (2021). Pembrolizumab plus chemotherapy versus chemotherapy alone for first-line treatment of advanced oesophageal cancer (KEYNOTE-590): A randomised, placebo-controlled, phase 3 study. Lancet.

[7] Okui J., Nagashima K., Matsuda S., Sato Y., Kawakubo H., Takeuchi M., Hirata K., Yamamoto S., Nomura M., Tsushima T. (2025). Investigating the synergistic effects of immunochemotherapy in esophageal squamous cell carcinoma. Esophagus.

[8] Khademi Z., Mahmoudi Z., Sukhorukov V.N., Jamialahmadi T., Sahebkar A. (2024). CRISPR/Cas9 Technology: A Novel Approach to Obesity Research. Curr. Pharm. Des..

[9] Bock C., Datlinger P., Chardon F., Coelho M.A., Dong M.B., Lawson K.A., Lu T., Maroc L., Norman T.M., Song B. (2022). High-content CRISPR screening. Nat. Rev. Methods Prim..

[10] Wang B., Xu Y., Wan A.H., Wan G., Wang Q.P. (2024). Integrating genome-wide CRISPR screens and in silico drug profiling for targeted antidote development. Nat. Protoc..

[11] Shalem O., Sanjana N.E., Hartenian E., Shi X., Scott D.A., Mikkelson T., Heckl D., Ebert B.L., Root D.E., Doench J.G. (2014). Genome-scale CRISPR-Cas9 knockout screening in human cells. Science.

[12] Ran F.A., Hsu P.D., Wright J., Agarwala V., Scott D.A., Zhang F. (2013). Genome engineering using the CRISPR-Cas9 system. Nat. Protoc..

[13] Ngan K.C., Lue N.Z., Lee C., Liau B.B. (2022). CRISPR-Suppressor Scanning for Systematic Discovery of Drug-Resistance Mutations. Curr. Protoc..

[14] MacLeod G., Rajakulendran N., Angers S. (2022). Identification of Drug Resistance Mechanisms Using Genome-Wide CRISPR-Cas9 Screens. Methods Mol. Biol..

[15] Selvakumar S.C., Preethi K.A., Ross K., Tusubira D., Khan M.W.A., Mani P., Rao T.N., Sekar D. (2022). CRISPR/Cas9 and next generation sequencing in the personalized treatment of Cancer. Mol. Cancer.

[16] Nguyen T.T., Ramsay L., Ahanfeshar-Adams M., Lajoie M., Schadendorf D., Alain T., Watson I.R. (2021). Mutations in the IFNgamma-JAK-STAT Pathway Causing Resistance to Immune Checkpoint Inhibitors in Melanoma Increase Sensitivity to Oncolytic Virus Treatment. Clin. Cancer Res..

[17] Li W., Koster J., Xu H., Chen C.H., Xiao T., Liu J.S., Brown M., Liu X.S. (2015). Quality control, modeling, and visualization of CRISPR screens with MAGeCK-VISPR. Genome Biol..

[18] Cui Y., Cheng X., Chen Q., Song B., Chiu A., Gao Y., Dawson T., Chao L., Zhang W., Li D. (2021). CRISP-view: A database of functional genetic screens spanning multiple phenotypes. Nucleic Acids Res..

[19] Nussinov R., Tsai C.J., Jang H. (2021). Anticancer drug resistance: An update and perspective. Drug Resist. Updat..

[20] Khan S.U., Fatima K., Aisha S., Malik F. (2024). Unveiling the mechanisms and challenges of cancer drug resistance. Cell Commun. Signal..

[21] Levatic J., Salvadores M., Fuster-Tormo F., Supek F. (2022). Mutational signatures are markers of drug sensitivity of cancer cells. Nat. Commun..

[22] Bernard E., Nannya Y., Hasserjian R.P., Devlin S.M., Tuechler H., Medina-Martinez J.S., Yoshizato T., Shiozawa Y., Saiki R., Malcovati L. (2020). Implications of TP53 allelic state for genome stability, clinical presentation and outcomes in myelodysplastic syndromes. Nat. Med..

[23] Ramalingam S.S., Vansteenkiste J., Planchard D., Cho B.C., Gray J.E., Ohe Y., Zhou C., Reungwetwattana T., Cheng Y., Chewaskulyong B. (2020). Overall Survival with Osimertinib in Untreated, EGFR-Mutated Advanced NSCLC. N. Engl. J. Med..

[24] Robichaux J.P., Le X., Vijayan R.S.K., Hicks J.K., Heeke S., Elamin Y.Y., Lin H.Y., Udagawa H., Skoulidis F., Tran H. (2021). Structure-based classification predicts drug response in EGFR-mutant NSCLC. Nature.

[25] Humeniuk R., Menon L.G., Mishra P.J., Gorlick R., Sowers R., Rode W., Pizzorno G., Cheng Y.C., Kemeny N., Bertino J.R. (2009). Decreased levels of UMP kinase as a mechanism of fluoropyrimidine resistance. Mol. Cancer Ther..

[26] Niu Y., Fan X., Wang Y., Lin J., Hua L., Li X., Qian R., Lu C. (2022). Genome-wide CRISPR Screening Reveals Pyrimidine Metabolic Reprogramming in 5-FU Chronochemotherapy of Colorectal Cancer. Front. Oncol..

[27] Shi L., Pan H., Liu Z., Xie J., Han W. (2017). Roles of PFKFB3 in cancer. Signal Transduct. Target. Ther..

[28] Kotowski K., Rosik J., Machaj F., Supplitt S., Wiczew D., Jablonska K., Wiechec E., Ghavami S., Dziegiel P. (2021). Role of PFKFB3 and PFKFB4 in Cancer: Genetic Basis, Impact on Disease Development/Progression, and Potential as Therapeutic Targets. Cancers.

[29] Deng X., Li D., Ke X., Wang Q., Yan S., Xue Y., Wang Q., Zheng H. (2021). Mir-488 alleviates chemoresistance and glycolysis of colorectal cancer by targeting PFKFB3. J. Clin. Lab. Anal..

[30] Jones B.C., Pohlmann P.R., Clarke R., Sengupta S. (2022). Treatment against glucose-dependent cancers through metabolic PFKFB3 targeting of glycolytic flux. Cancer Metastasis Rev..

[31] Lypova N., Dougherty S.M., Lanceta L., Chesney J., Imbert-Fernandez Y. (2021). PFKFB3 Inhibition Impairs Erlotinib-Induced Autophagy in NSCLCs. Cells.

[32] Yan S., Li Q., Li S., Ai Z., Yuan D. (2022). The role of PFKFB3 in maintaining colorectal cancer cell proliferation and stemness. Mol. Biol. Rep..

[33] Deng J., Cheng Y., Li H., He X., Yu S., Ma J., Li X., Chen J., Xiao H., Guan H. (2024). PFKFB3 facilitates cell proliferation and migration in anaplastic thyroid carcinoma via the WNT/beta-catenin signaling pathway. Endocrine.

[34] Marcucci F., Rumio C. (2021). Glycolysis-induced drug resistance in tumors-A response to danger signals?. Neoplasia.

[35] Sethy C., Kundu C.N. (2021). 5-Fluorouracil (5-FU) resistance and the new strategy to enhance the sensitivity against cancer: Implication of DNA repair inhibition. Biomed. Pharmacother..

[36] Ohashi S., Kikuchi O., Nakai Y., Ida T., Saito T., Kondo Y., Yamamoto Y., Mitani Y., Nguyen Vu T.H., Fukuyama K. (2020). Synthetic Lethality with Trifluridine/Tipiracil and Checkpoint Kinase 1 Inhibitor for Esophageal Squamous Cell Carcinoma. Mol. Cancer Ther..

[37] Federica G., Michela C., Giovanna D. (2024). Targeting the DNA damage response in cancer. MedComm.

[38] Drew Y., Zenke F.T., Curtin N.J. (2025). DNA damage response inhibitors in cancer therapy: Lessons from the past, current status and future implications. Nat. Rev. Drug Discov..

[39] Okita N., Minato S., Ohmi E., Tanuma S., Higami Y. (2012). DNA damage-induced CHK1 autophosphorylation at Ser296 is regulated by an intramolecular mechanism. FEBS Lett..

[40] Tantawy S.I., Timofeeva N., Sarkar A., Gandhi V. (2023). Targeting MCL-1 protein to treat cancer: Opportunities and challenges. Front. Oncol..

[41] Hafezi S., Rahmani M. (2021). Targeting BCL-2 in Cancer: Advances, Challenges, and Perspectives. Cancers.

[42] Zhang J., Wang Y., Yin C., Gong P., Zhang Z., Zhao L., Waxman S., Jing Y. (2022). Artesunate improves venetoclax plus cytarabine AML cell targeting by regulating the Noxa/Bim/Mcl-1/p-Chk1 axis. Cell Death Dis..

[43] Long S., Wang J., Weng F., Pei Z., Zhou S., Sun G., Xiang D. (2022). ECM1 regulates the resistance of colorectal cancer to 5-FU treatment by modulating apoptotic cell death and epithelial-mesenchymal transition induction. Front. Pharmacol..

[44] Ma J., Ma Y., Chen S., Guo S., Hu J., Yue T., Zhang J., Zhu J., Wang P., Chen G. (2021). SPARC enhances 5-FU chemosensitivity in gastric cancer by modulating epithelial-mesenchymal transition and apoptosis. Biochem. Biophys. Res. Commun..

[45] Tiu Y.C., Gong L., Zhang Y., Luo J., Yang Y., Tang Y., Lee W.M., Guan X.Y. (2022). GLIPR1 promotes proliferation, metastasis and 5-fluorouracil resistance in hepatocellular carcinoma by activating the PI3K/PDK1/ROCK1 pathway. Cancer Gene Ther..

[46] Matsuura N., Tanaka K., Yamasaki M., Yamashita K., Saito T., Makino T., Yamamoto K., Takahashi T., Kurokawa Y., Nakajima K. (2021). NOTCH3 limits the epithelial-mesenchymal transition and predicts a favorable clinical outcome in esophageal cancer. Cancer Med..

[47] Saitoh M. (2023). Transcriptional regulation of EMT transcription factors in cancer. Semin. Cancer Biol..

[48] Ang H.L., Mohan C.D., Shanmugam M.K., Leong H.C., Makvandi P., Rangappa K.S., Bishayee A., Kumar A.P., Sethi G. (2023). Mechanism of epithelial-mesenchymal transition in cancer and its regulation by natural compounds. Med. Res. Rev..

[49] Kim S., Yao J., Suyama K., Qian X., Qian B.Z., Bandyopadhyay S., Loudig O., De Leon-Rodriguez C., Zhou Z.N., Segall J. (2014). Slug promotes survival during metastasis through suppression of Puma-mediated apoptosis. Cancer Res..

[50] Assani G., Zhou Y. (2019). Effect of modulation of epithelial-mesenchymal transition regulators Snail1 and Snail2 on cancer cell radiosensitivity by targeting of the cell cycle, cell apoptosis and cell migration/invasion. Oncol. Lett..

[51] Zhang K., Zhang S., Jiao X., Wang H., Zhang D., Niu Z., Shen Y., Lv L., Zhou Y. (2011). Slug regulates proliferation and invasiveness of esophageal adenocarcinoma cells in vitro and in vivo. Med. Oncol..

[52] Jumper J., Evans R., Pritzel A., Green T., Figurnov M., Ronneberger O., Tunyasuvunakool K., Bates R., Zidek A., Potapenko A. (2021). Highly accurate protein structure prediction with AlphaFold. Nature.

[53] Jaiswal A.S., Dutta A., Srinivasan G., Yuan Y., Zhou D., Shaheen M., Sadideen D.T., Kirby A., Williamson E.A., Gupta Y.K. (2023). TATDN2 resolution of R-loops is required for survival of BRCA1-mutant cancer cells. Nucleic Acids Res..

[54] Zhang J., Pei J., Durham J., Bos T., Cong Q. (2022). Computed cancer interactome explains the effects of somatic mutations in cancers. Protein Sci..

[55] Hardison K.L., Hawk T.M., Bouley R.A., Petreaca R.C. (2022). KAT5 histone acetyltransferase mutations in cancer cells. microPubl. Biol..

[56] Ren F., Ding X., Zheng M., Korzinkin M., Cai X., Zhu W., Mantsyzov A., Aliper A., Aladinskiy V., Cao Z. (2023). AlphaFold accelerates artificial intelligence powered drug discovery: Efficient discovery of a novel CDK20 small molecule inhibitor. Chem. Sci..

[57] Doench J.G., Fusi N., Sullender M., Hegde M., Vaimberg E.W., Donovan K.F., Smith I., Tothova Z., Wilen C., Orchard R. (2016). Optimized sgRNA design to maximize activity and minimize off-target effects of CRISPR-Cas9. Nat. Biotechnol..

