# Supplementary material for: CRISPR/Cas9 Screening Highlights PFKFB3 Gene as a Major Contributor to 5-Fluorouracil Resistance in Esophageal Cancer
Source: Cancers (Basel). 2025 May 12;17(10):1637. doi: 10.3390/cancers17101637 (PMC12109790; doi:10.3390/cancers17101637)
Supplement: Supplementary file 1 [file cancers-17-01637-s001.zip › Xue et al. Supplementary data.pdf]

Supplementary Figures

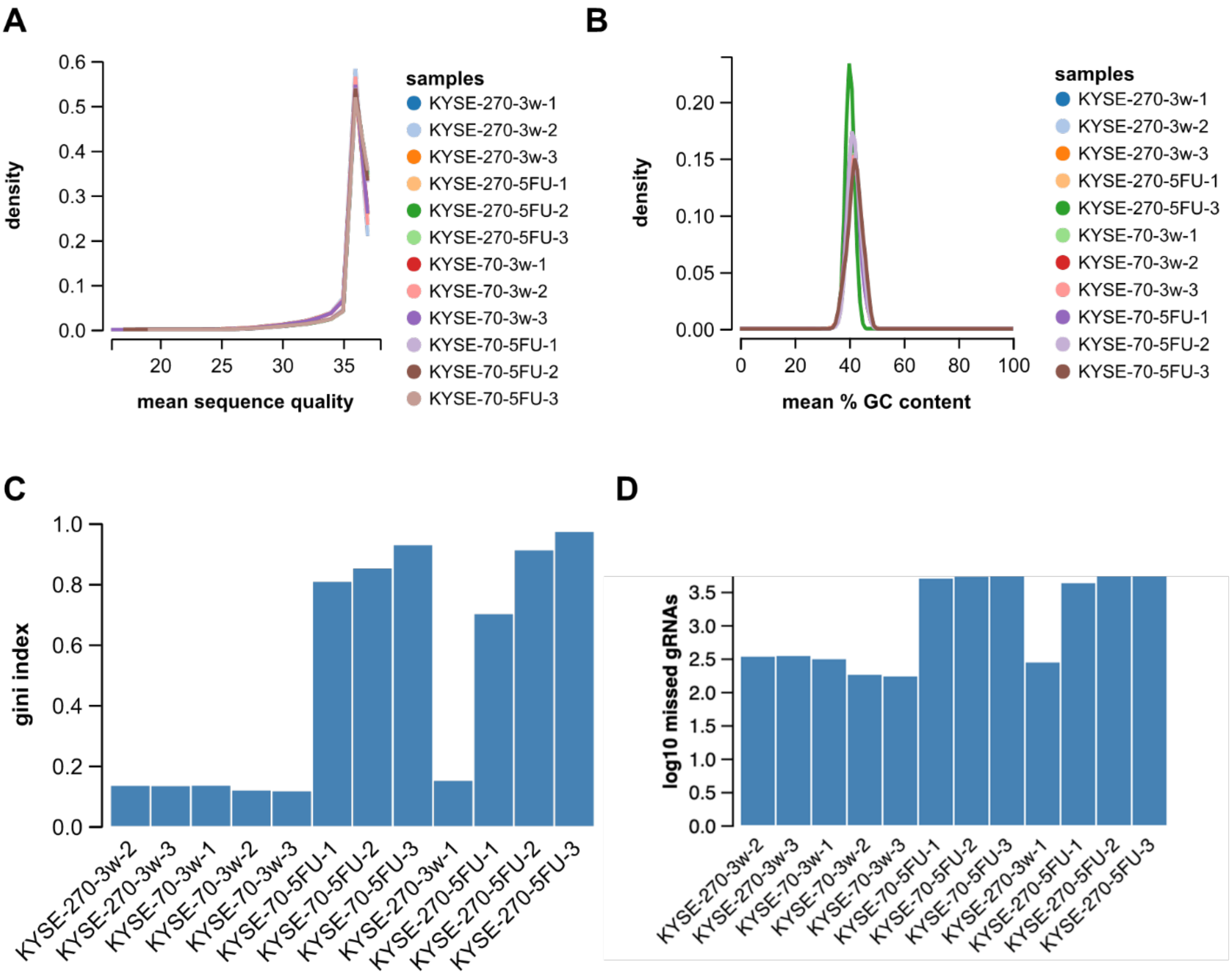

Fig. S1: The quality control (QC) view of MAGeCK-VISPR. (A) Distribution of per-read mean base quality for each sample. The peak should be beyond 30. **(B)** GC content distribution of the sequencing reads. Similar distribution for all samples from same library. **(C)** Gini Index for each sample. This measures inequality between read count. 1.0 means maximum inequality, 0.0 corresponds to having the same read count for all sgRNAs. **(D)** The number of zero count sgRNAs per sample.

A

KYSE-70-WT

CCCCTTCTTTCGCCAGGTAGCTTTTGACATCTCTCAAGGCA--GCTAAGGC

KYSE-70-NC

CCCCTTCTTTCGCCAGGTAGCTTTTGACATCTCTCAAGGCA--GCTAAGGC

KYSE-70-sg1-8

CCCCTTCTTTCGCCAGGTAGCTTTTGACATCTCTCAAGGCAGAGCTAAGGC

sgRNA1-reverse .............................GACATCTCTCAAGGCAGCTA............

B

KYSE-70-WT

CGCCTCCCAGGACTCACCGCGTC-GATCTCATTGAGCGCCTTCCACTGCTC

KYSE-70-NC

CGCCTCCCAGGACTCACCGCGTC-GATCTCATTGAGCGCCTTCCACTGCTC

KYSE-70-sg2-13

CGCCTCCCAGGACTCACCGCGTCCGATCTCATTGAGCGCCTTCCACTGCTC

sgRNA2-reverse ..................CGTCGATCTCATTGAGCGCC....................

C

KYSE-150-WT

CGCCAGGTAGCTTTTGACATCTCTCAAGGCAGCTAAGGCACATTGCCTGGAA

KYSE-150-NC

CGCCAGGTAGCTTTTGACATCTCTCAAGGCAGCTAAGGCACATTGCCTGGAA

Mutation Type 1:

KYSE-150-sg1-19

CGCCAGG-----------------------------------43bp--AA

sgRNA1-reverse ..........GACATCTCTCAAGGCAGCTA...................................

Mutation Type 2:

KYSE-150-sg1-19

CGCCAGGTAGCTTTTGACATCTCTC-----------------------124bp------------------------

D

KYSE-150-WT

CGCCTCCCAGGACTCACCGCGTC-GATCTCATTGAGCGCCTTCCACTGCTC

KYSE-150-NC

CGCCTCCCAGGACTCACCGCGTC-GATCTCATTGAGCGCCTTCCACTGCTC

KYSE-150-sg2-7

CGCCTCCCAGGACTCACCGCGTCCGATCTCATTGAGCGCCTTCCACTGCTC

sgRNA2-reverse ................CGTCGATCTCATTGAGCGCC.........................

E

KYSE-270-WT

CCCCTTCTTTCGCCAGGTAGCTTTTGACATCTCTCAAGGCAGCTAAGGC

KYSE-270-NC

CCCCTTCTTTCGCCAGGTAGCTTTTGACATCTCTCAAGGCAGCTAAGGC

KYSE-270-sg1-19

CCCCTTCTTTCGCCAGGTAGCTTTTGACATCTCTCAA----5bp----CTAAGGC

sgRNA1-reverse ….....………………… GACATCTCTCAAGGCAGCTA……….

F

KYSE-270-WT

CGCCTCCCAGGACTCACCGCGTC-GATCTCATTGAGCGCCTTCCACTGCTC

KYSE-270-NC

CGCCTCCCAGGACTCACCGCGTC-GATCTCATTGAGCGCCTTCCACTGCTC

Mutation Type 1:

KYSE-270-sg2-11

CGCCTCCCAGGACTCACCGCGTCCGATCTCATTGAGCGCCTTCCACTGCTC

sgRNA2-reverse ....…. CGTCGATCTCATTGAGCGCC……………………

Mutation Type 2:

CGCCTCCCAGGACTCACCGCG-1bp-CGATCTCATTGAGCGCCTTCCACTGCTC

Fig. S2: Sanger sequencing validation of mutations in KYSE-70, KYSE-270 and KYSE-150 cell lines. (A-F) Presents the sequencing confirmation of mutation in PFKFB3 knockout cell lines. Sequence alignments show the presence of specific mutation (e.g., insertion or deletion) compared to the wild-type and negative control sequence. The yellow markers correspond to the sequences of the sgRNAs, the red markers refer to the inserted fragments, and the grey markers are the deleted fragments.

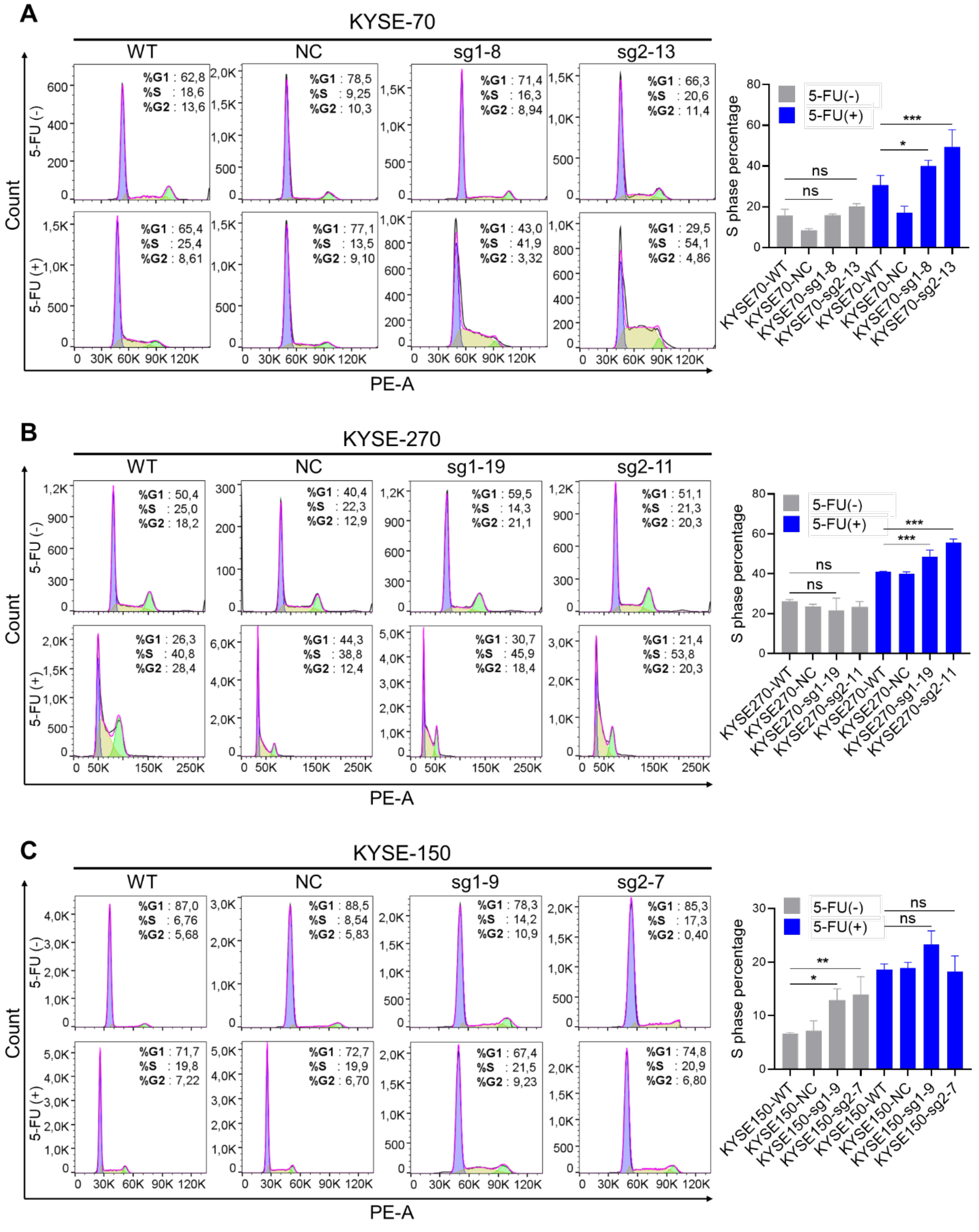

Fig. S3: The impact of the PFKFB3 on cell cycle in human esophageal cancer cell lines. (A) Flow cytometry analysis of cell cycle distribution in the KYSE-70 cell line showed an increase in the proportion of cells in the S phase following PFKFB3 knockout after 5-FU treatment. **(B)** The S

phase was significantly prolonged in PFKFB3 knockout KYSE-270 cells after 5-FU treatment. **(C)** The S phase was significantly prolonged in PFKFB3 knockout KYSE-150 cells before 5-FU treatment, while no significant changes were observed in the S phase after 5-FU treatment. The data are presented as the mean of three independent experiments. The statistical significance of the results was determined using one-way ANOVA, with the following p-values: * $P < 0.05$; ** $P < 0.01$ and *** $P < 0.001$.
