# Supplementary material for: CRISPR/Cas9 Screening Highlights PFKFB3 Gene as a Major Contributor to 5-Fluorouracil Resistance in Esophageal Cancer
Source: Cancers (Basel). 2025 May 12;17(10):1637. doi: 10.3390/cancers17101637 (PMC12109790; doi:10.3390/cancers17101637)
Supplement: Supplementary file 1 [file cancers-17-01637-s001.zip › Xue et al. Western blots_ original figures.pdf]

Full length uncropped original western blots figures

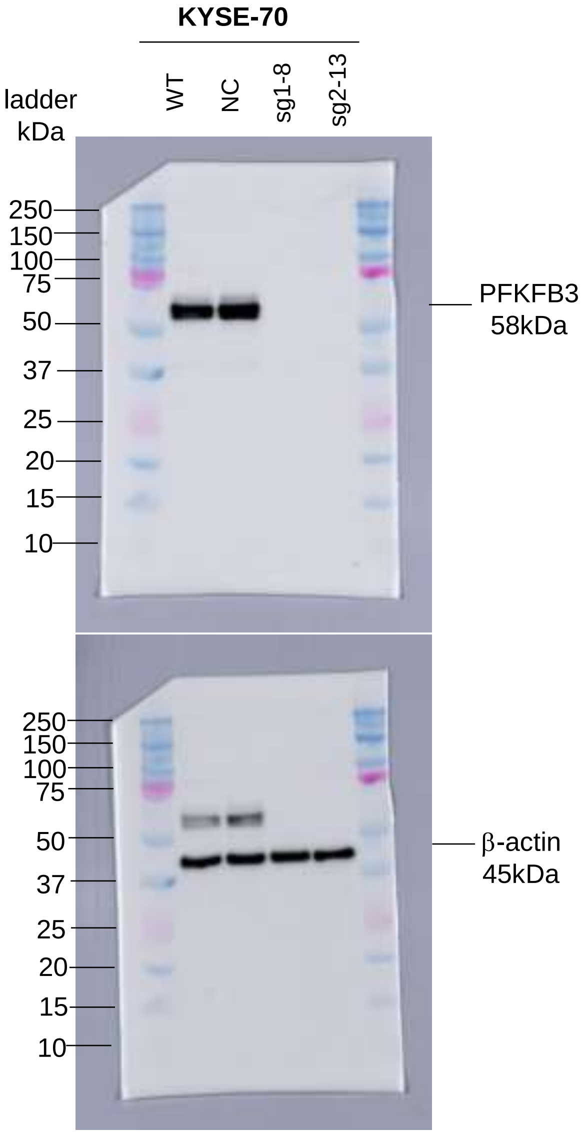

Figure 1C

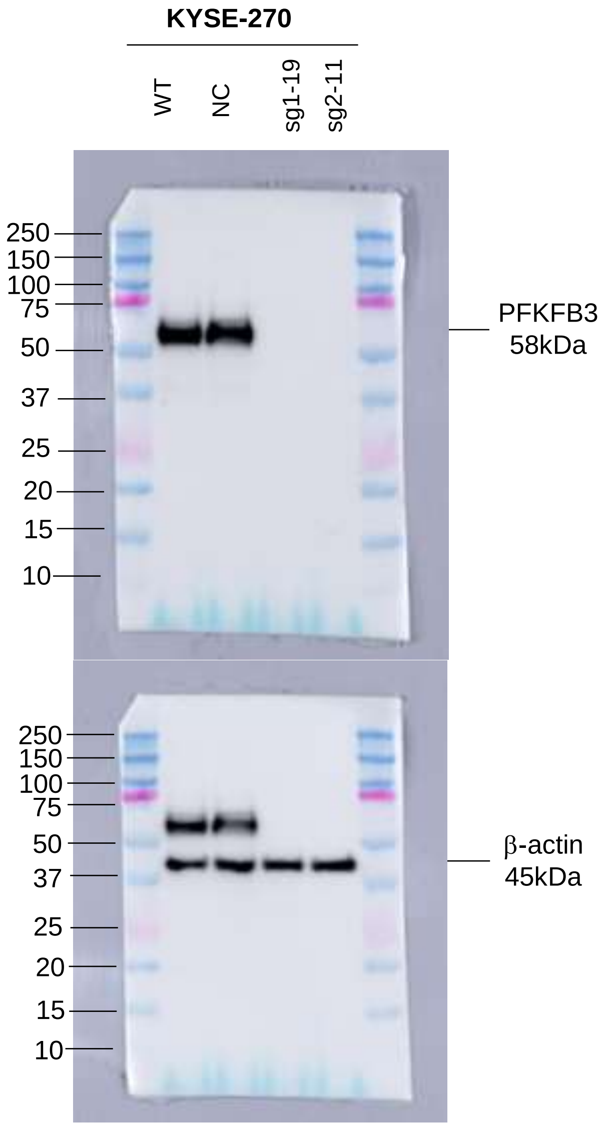

Figure 1D

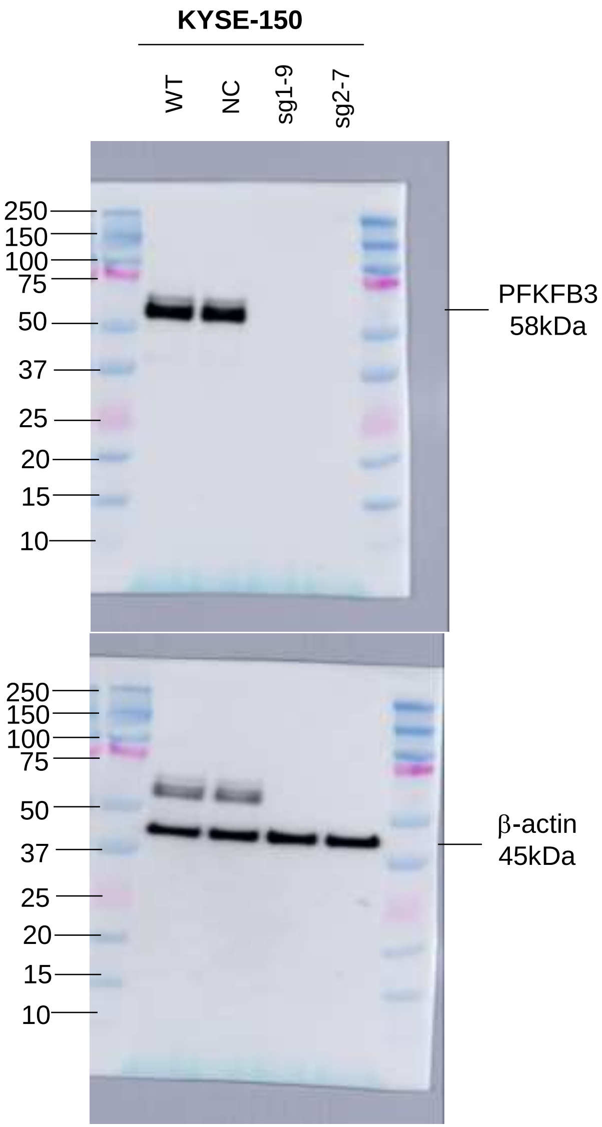

Figure 1E

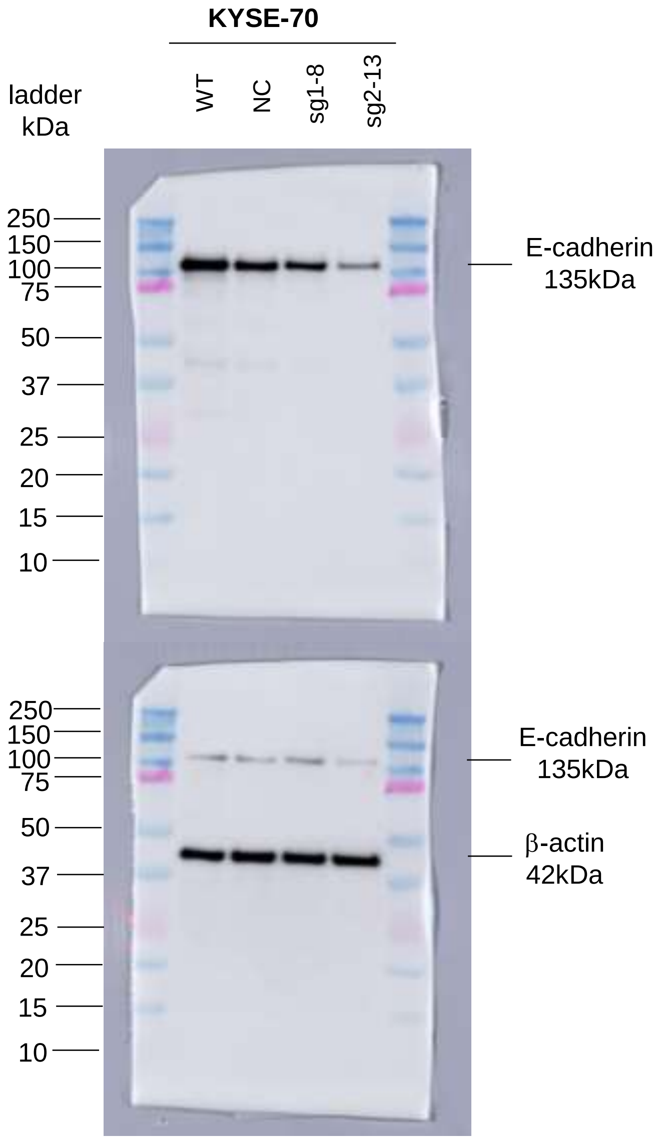

Figure 4A

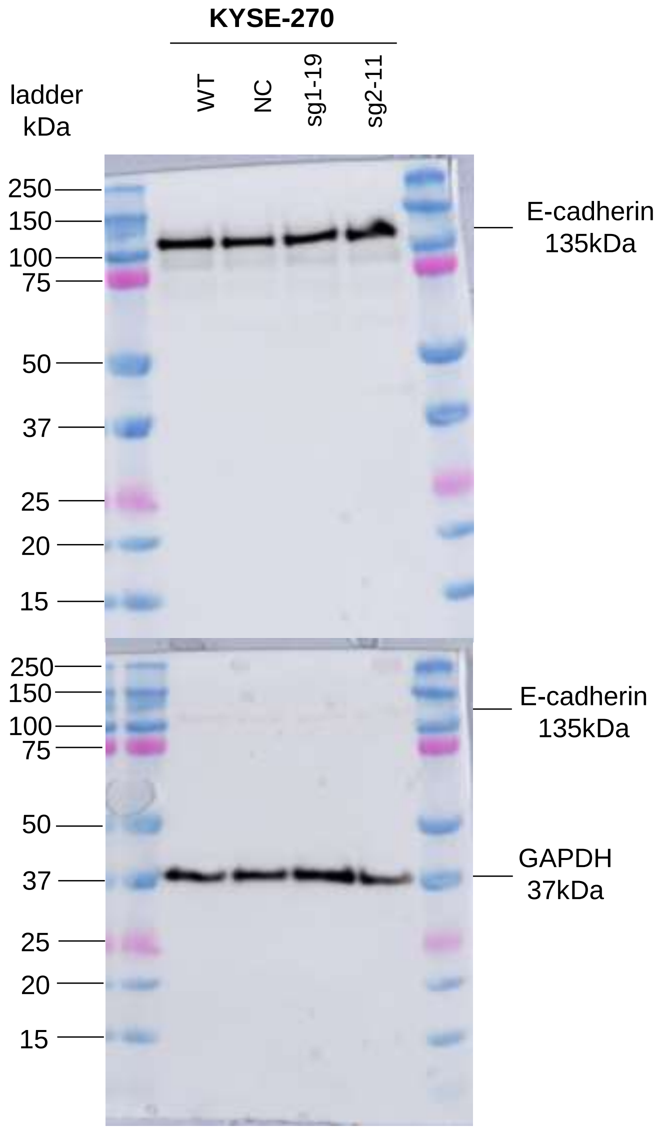

Figure 4B

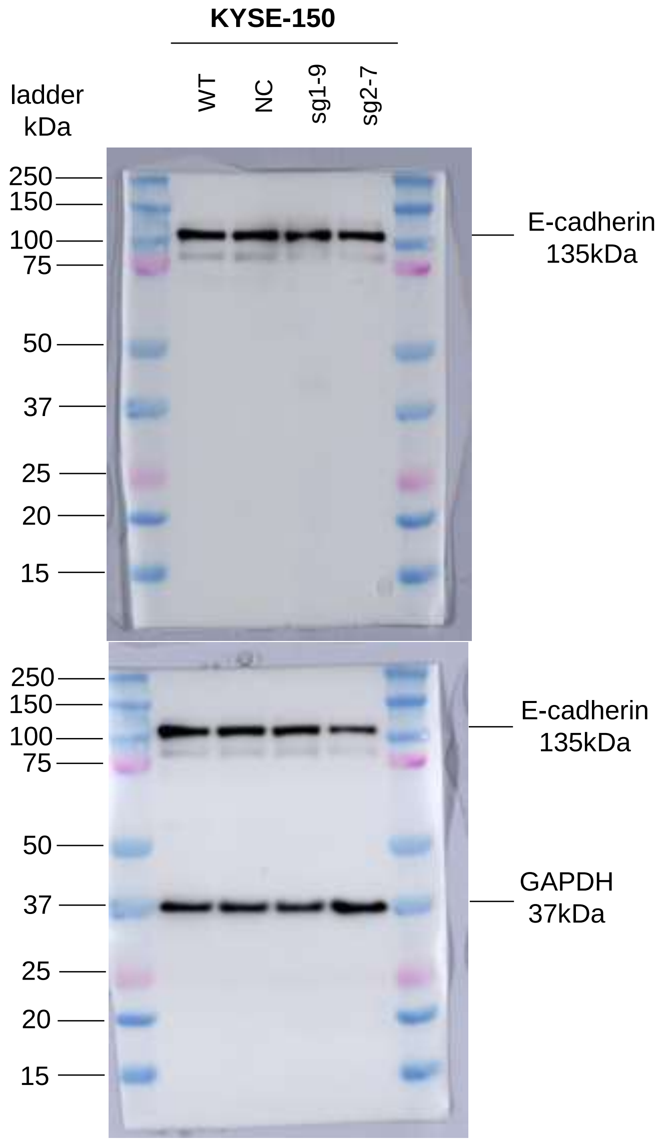

Figure 4C

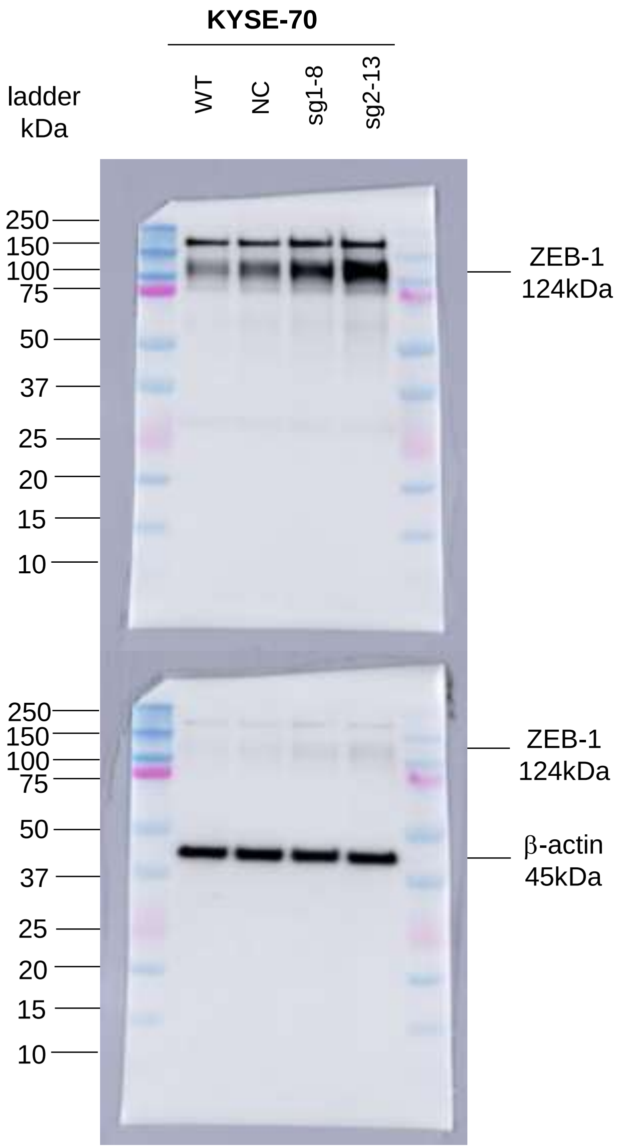

Figure 4D

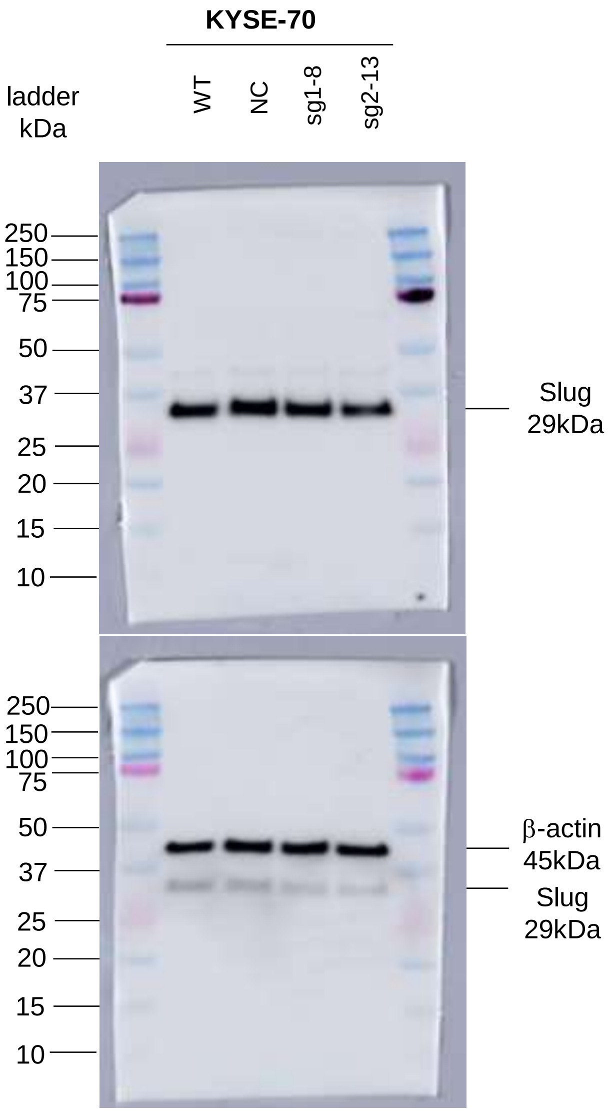

Figure 4D

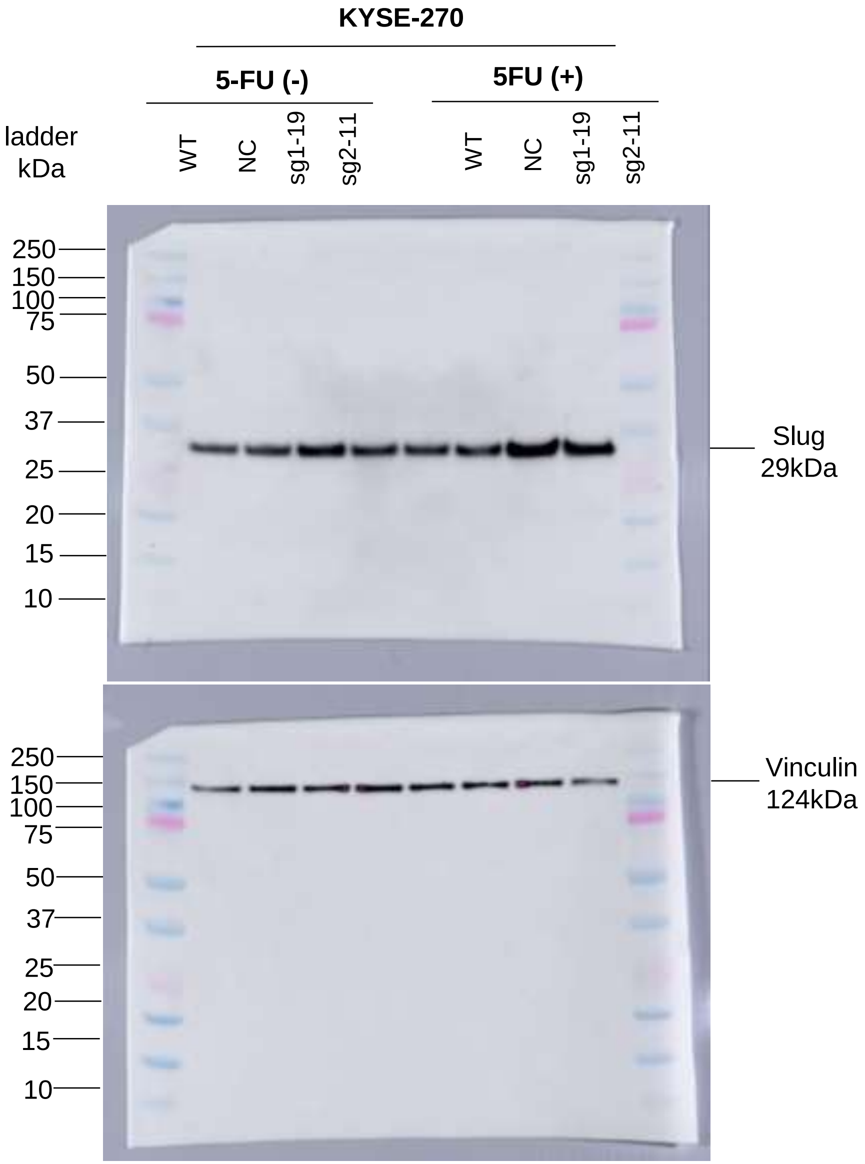

Figure 5A

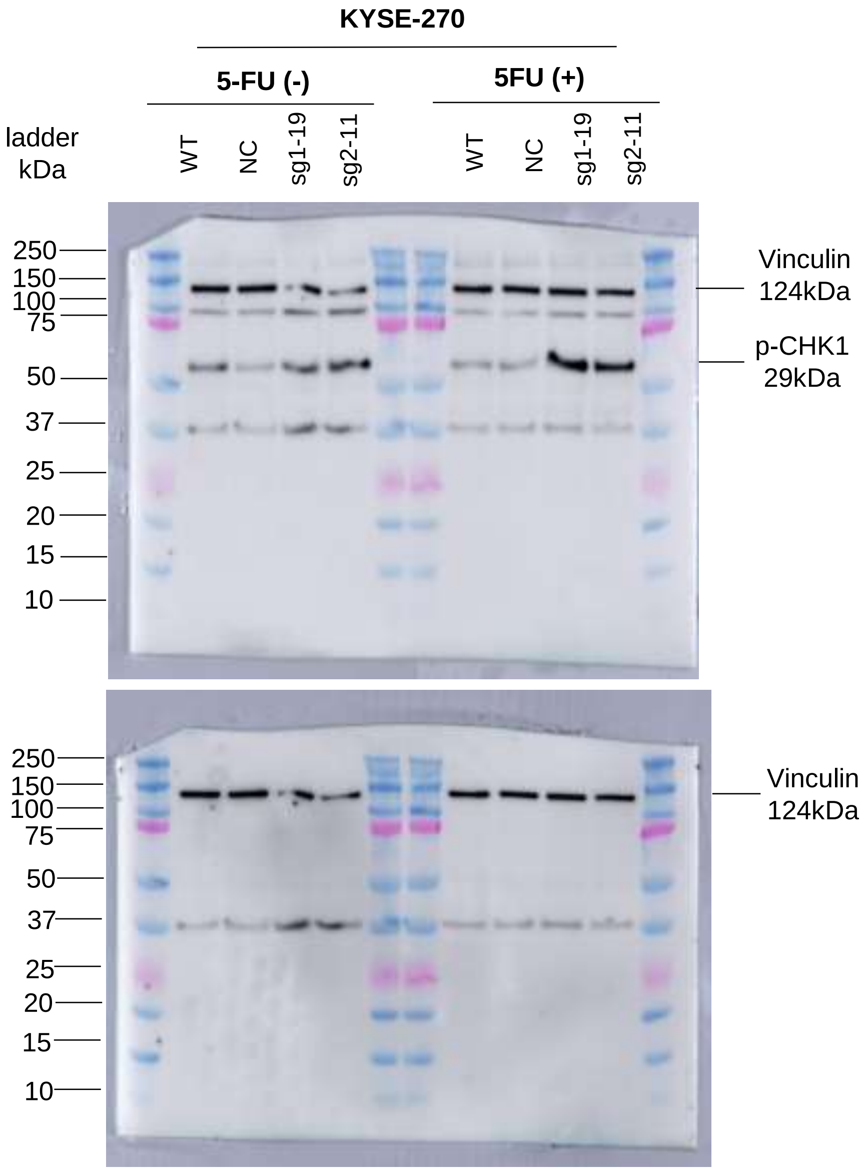

Figure 5A

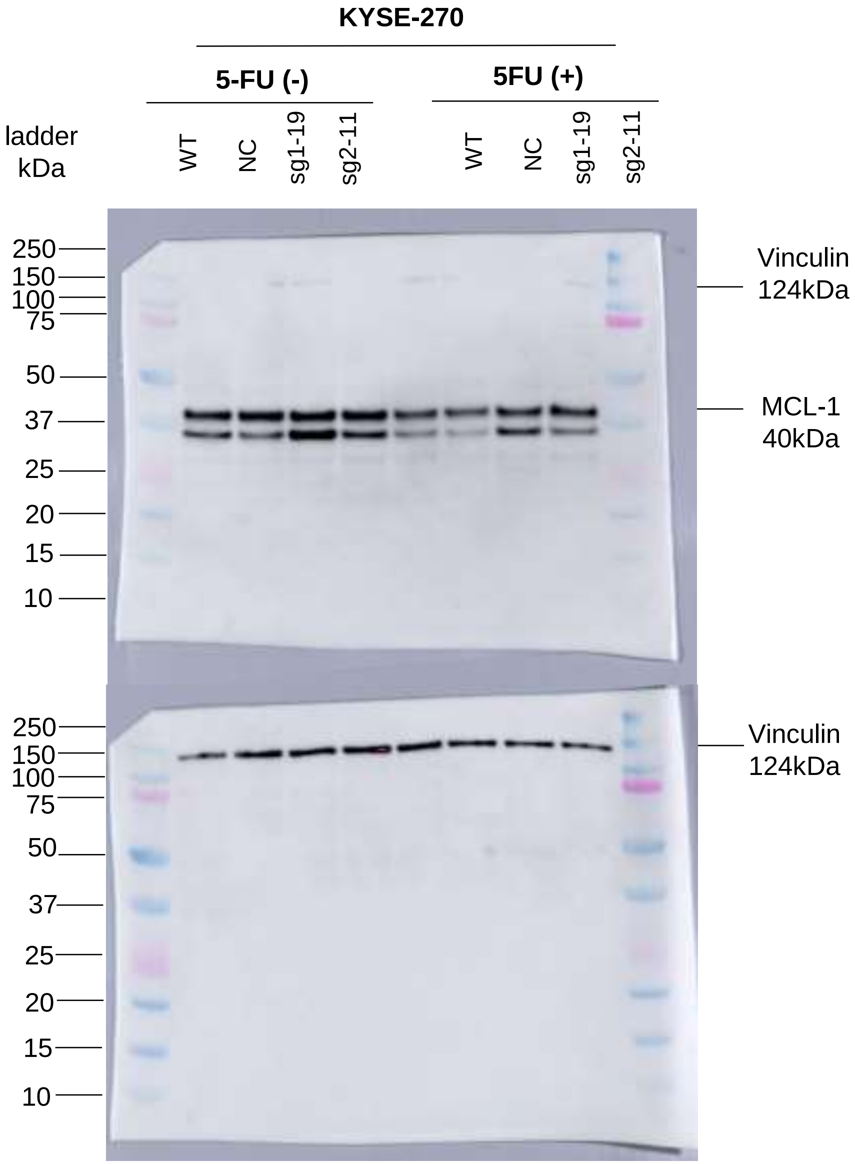

Figure 5A

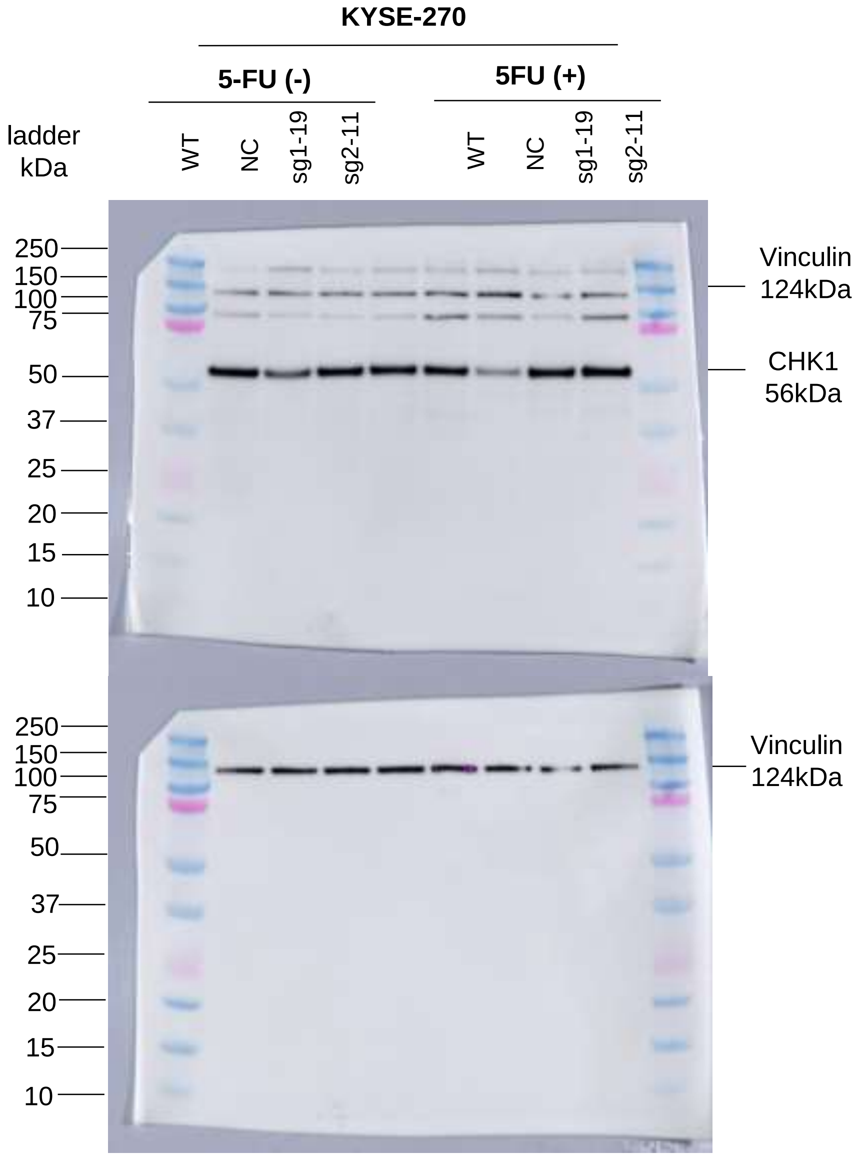

Figure 5A

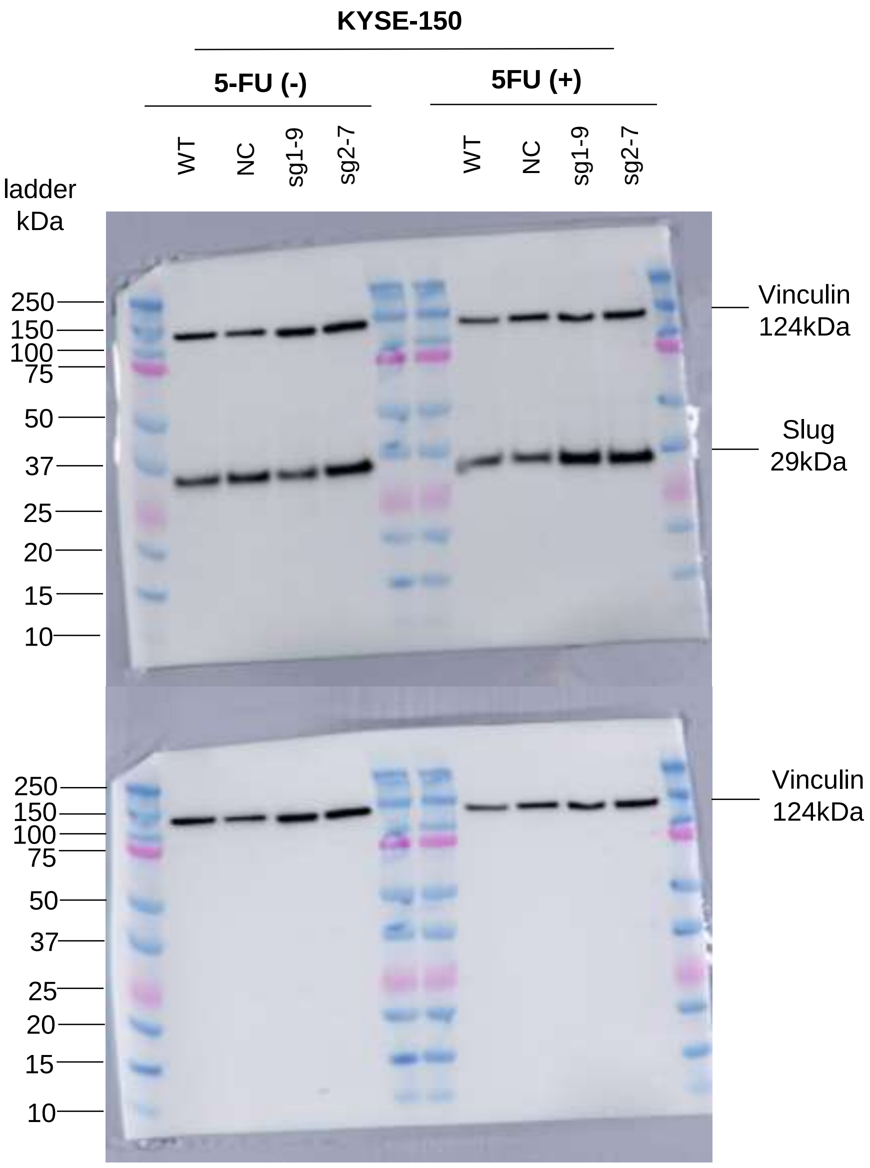

Figure 5B

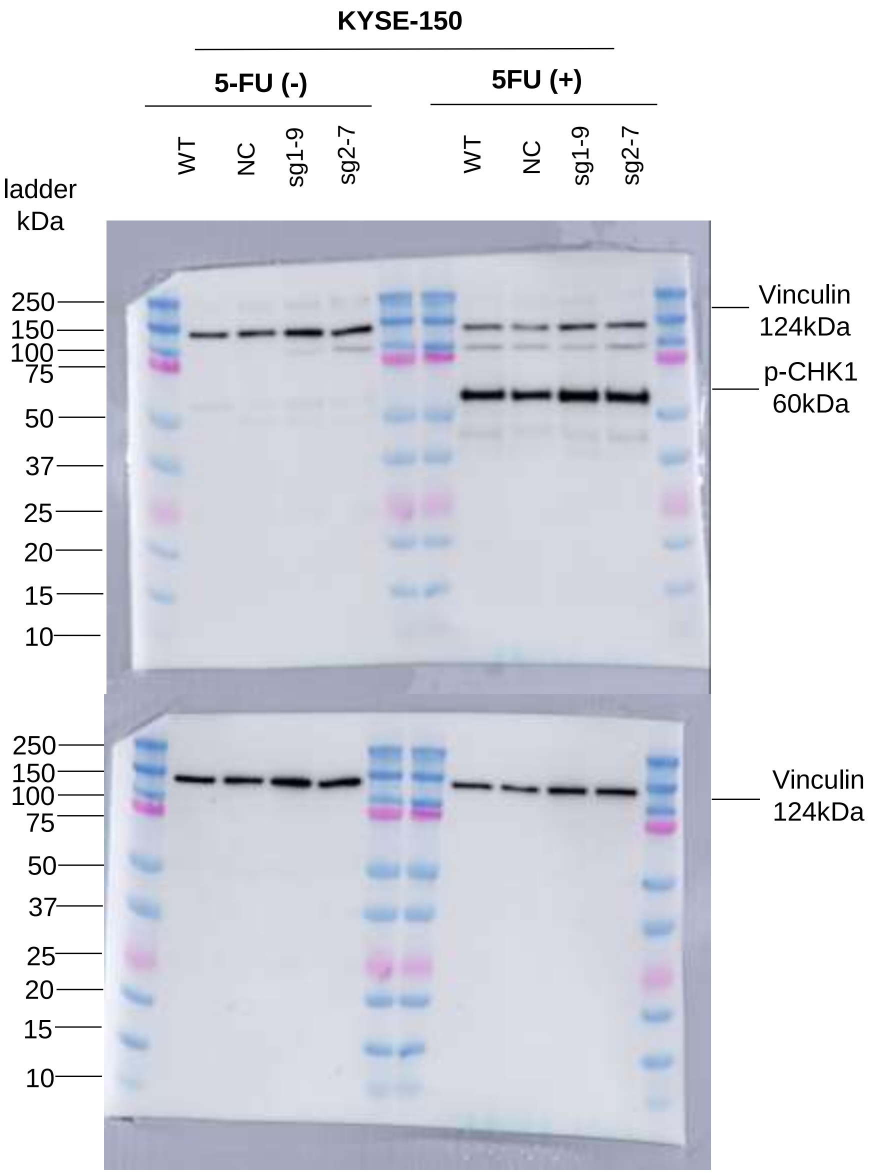

Figure 5B

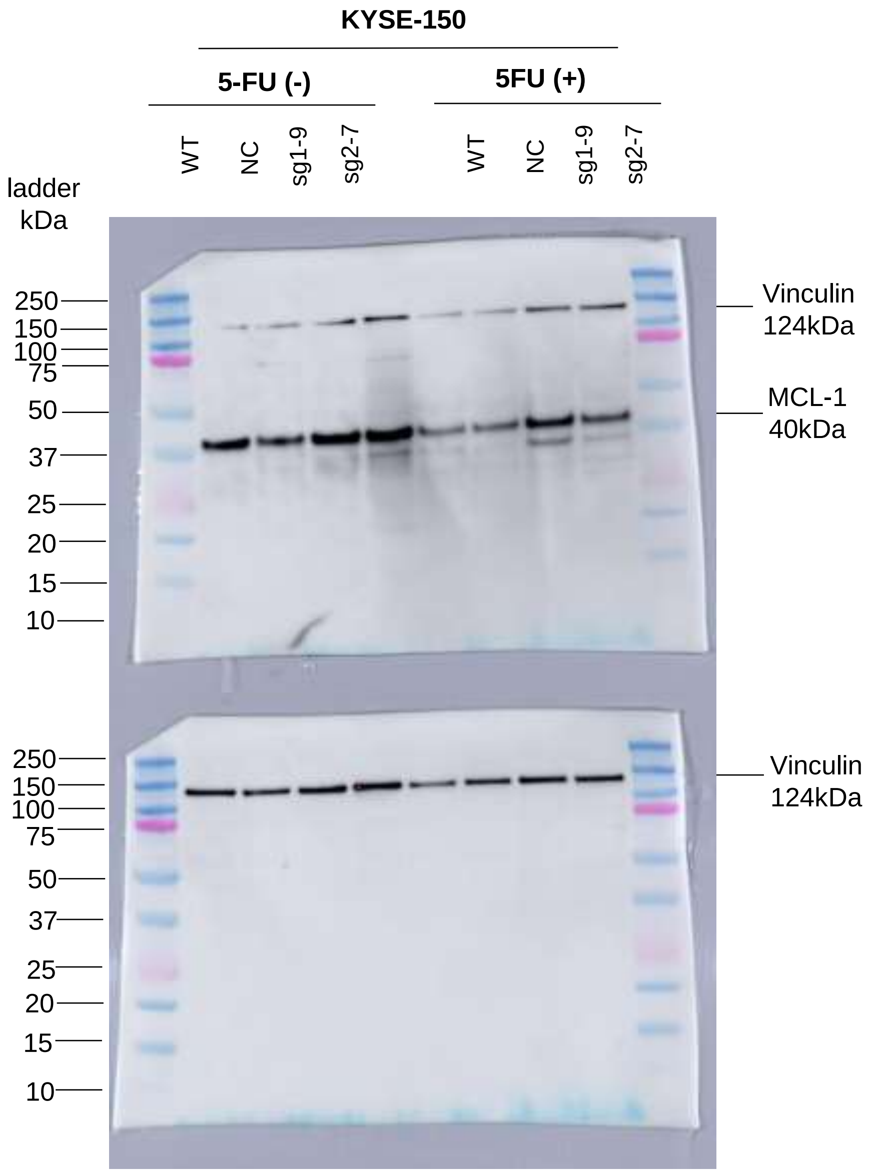

Figure 5B

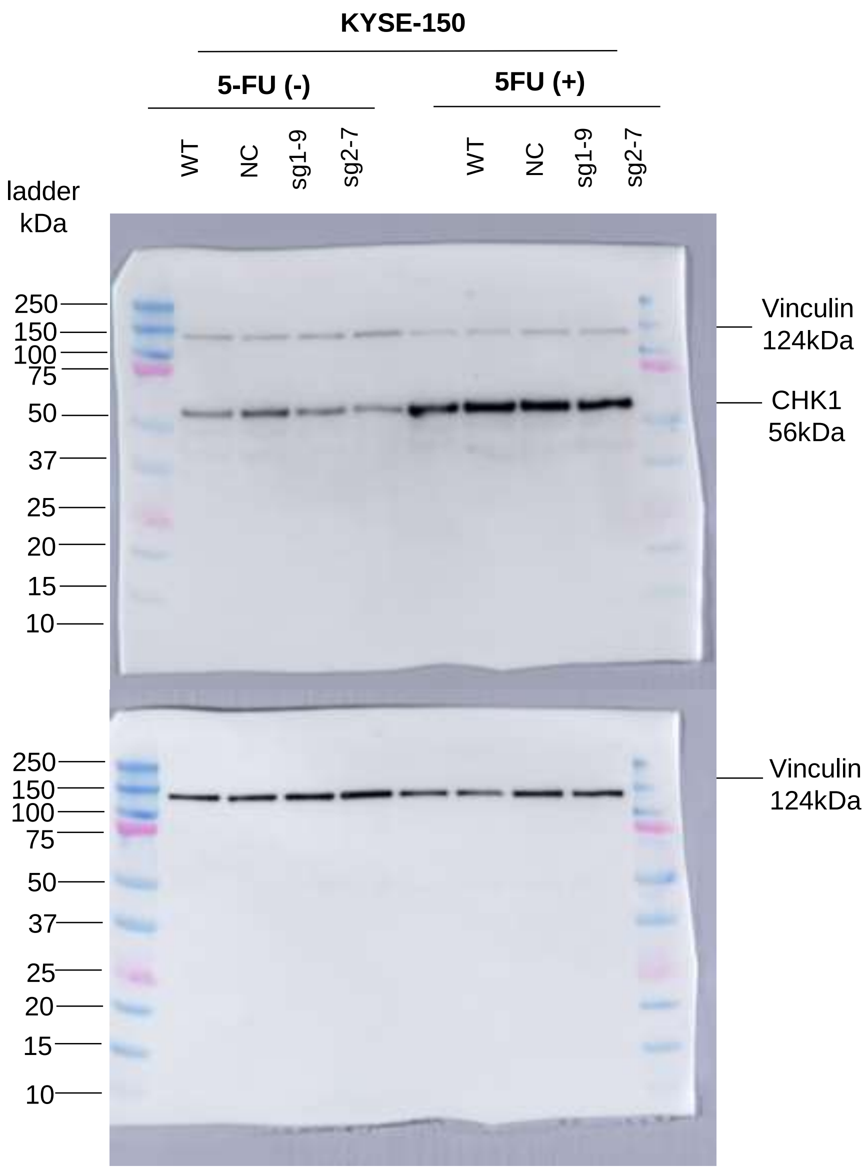

Figure 5B
